# Circulating exosomal microRNA profiles in migraine patients receiving acupuncture treatment: A placebo-controlled clinical trial

**DOI:** 10.3389/fnmol.2022.1098766

**Published:** 2023-01-10

**Authors:** Lu Liu, Wenchuan Qi, Yanan Wang, Xixiu Ni, Shan Gao, Ziyang Zhou, Daohong Chen, Zhenxi He, Mingsheng Sun, Ziwen Wang, Dingjun Cai, Ling Zhao

**Affiliations:** ^1^Acupuncture and Tuina School, Chengdu University of Traditional Chinese Medicine, Chengdu, Sichuan, China; ^2^Acupuncture and Chronobiology Key Laboratory of Sichuan Province, Chengdu, Sichuan, China

**Keywords:** acupuncture, migraine, biomarker, exosome, miRNA

## Abstract

**Background:**

Acupuncture has a long history of being used in Chinese medicine for the treatment of migraine. However, molecular biomarkers for diagnosis and prognosis of migraine and its treatment are lacking. This study aimed to explore whether acupuncture could regulate differentially expressed exosomal miRNAs between patients with migraine without aura (MWoA) and healthy controls (HCs) and to identify diagnostic biomarkers that helped differentiate MWoA patients from HCs and identify prognostic biomarkers that helped to predict the effect of acupuncture.

**Methods:**

Here, we isolated serum exosomes from patients with MWoA and HCs before and after true and sham acupuncture treatment. Then, small RNA sequencing and bioinformatics analysis were performed to screen out key miRNAs specifically responding to acupuncture treatment. Pearson’s correlation analysis was used to evaluate the correlation between miRNAs and clinical phenotypes. Finally, we applied a machine learning method to identify diagnostic biomarkers of MWoA patients and identify prognostic biomarkers that helped to predict the effect of acupuncture.

**Results:**

Small RNA sequencing identified 68 upregulated and 104 downregulated miRNAs in MWoA patients compared to those in HCs. Further, we identified eight upregulated and four downregulated miRNAs in migraine patients after true acupuncture treatment (trAMWoA), but not in the sham acupuncture treatment (shAMWoA) or HC group. Among them, hsa-miR-378a-5p was positively correlated with time unable to work, study, or do housework due to migraine (*p* < 0.05), whereas hsa-miR-605-3p was negatively correlated with the restrictive subscale of the migraine-specific quality of life questionnaire (MSQ) (*p* < 0.05). We then evaluated the diagnostic and prognostic potential of these 12 miRNAs in patients with MWoA. The combination of serum levels of exosomal hsa-miR-369-5p, hsa-miR-145-5p, and hsa-miR-5,010-3p could serve as diagnostic and prognostic biomarkers for MWoA patients following acupuncture treatment.

**Conclusion:**

This is the first study on the serum exosomal miRNA profiles of migraineurs before and after acupuncture treatment. Our results improve our understanding of the molecular functions of miRNAs in MWoA. More importantly, they expand our view of evaluating the clinical outcomes of migraine patients treated with acupuncture, using exosomal RNA markers.

**Clinical Trial Registration:**

Chinese Clinical Trial Registry, ChiCTR2000034417, July 2020.

## 1. Introduction

Migraine, the sixth most common condition in the world and the second most disabling condition (GBD 2016 Neurology Collaborators, 2018), is a chronic neurological condition characterized by recurrent headaches that are typically unilateral, throbbing, and associated with photophobia, nausea, and vomiting (Headache classification Committee of the International Headache Society (IHS), 2018). The latest Global Burden of Disease Study showed that 1.25 billion people suffered from migraine in 2017 (GBD 2016 Neurology Collaborators, 2018). Despite the improvement in migraine medications (Farri et al., 2012; Ou et al., 2020), patients are often still unsatisfied because of disturbing adverse events (Tfelt-Hansen et al., 2000; Nagy et al., 2011; Ong and De Felice, 2018). Therefore, non-pharmacological therapies with fewer adverse effects are required. Evidences from clinical trials and systematic reviews demonstrate that acupuncture has emerged as an effective non-pharmacological therapy for migraine that lacks serious side effects (Streng et al., 2006; Li et al., 2012; Foroughipour et al., 2014; Linde et al., 2016; Zhao et al., 2017; Li et al., 2019; Giovanardi et al., 2020; Ou et al., 2020; Xu et al., 2020; Zhang et al., 2020).

The diagnosis of migraine is solely based on the criteria established by the International Classification of Headache Disorders (ICHD-3) (Headache classification Committee of the International Headache Society (IHS), 2018). Relief from migraine is measured by self-reported clinical symptoms, including the frequency of migraine attacks, mean duration of migraine attacks, and visual analog scale (VAS) rating of pain (Nagy et al., 2011; Li et al., 2012; GBD 2016 Neurology Collaborators, 2018). Currently, there can be partial subjectivity when making migraine diagnoses and substantial difficulty when attempting to prognosticate patient outcomes and predict responses to migraine therapies, which has fueled research efforts to establish migraine-specific diagnostic and prognostic biomarkers for precision medicine approaches. Researchers have identified migraine biomarkers using genetics (Gormley et al., 2016), neuroimaging studies (Schwedt et al., 2015; Mu et al., 2020; Tu et al., 2020), and biochemistry (Cernuda-Morollón et al., 2013; Iyengar et al., 2017). Moreover, numerous studies have been conducted to identify genetic, neuroimaging, and blood prognostic markers in migraine patients (Cernuda-Morollón et al., 2014; Liu H.Y. et al., 2017; Liu Y.Y. et al., 2017; Liu et al., 2017a,b; Domínguez et al., 2018; Moreno-Mayordomo et al., 2019). Acupuncture has been effective in treating migraine; however, to date, only neuroimaging biomarkers have been used to predict acupuncture efficacy (Liu H.Y. et al., 2017; Liu Y.Y. et al., 2017; Liu et al., 2017a,b, 2019; Yang et al., 2020). There is still a scarcity of blood and genetic biomarkers that can be used to predict acupuncture efficacy.

Exosomes are micro vesicles that range from 30 to 100 nm in size and are secreted by all living cells (Simpson et al., 2009). They specialize in long-distance intracellular communication and facilitate the transfer of nucleic acids, such as messenger RNAs and miRNAs, for subsequent expression in target cells in a highly regulated and efficient manner (Madison and Okeoma, 2015; Ellwanger et al., 2017). Exosomal miRNAs can serve as valuable non-invasive biomarkers that can be objectively measured to assist in migraine diagnosis, improve the understanding of its pathogenesis, and determine its potential response to treatment (Nicholas, 2013).

We have previously reported the superior effect of true acupuncture (TA) compared to sham acupuncture (SA) in treating migraine (Zhao et al., 2017). Furthermore, we found that the inter-individual variability of brain structure could predict the outcomes of an 8-week sham acupuncture treatment in migraine without aura (MWoA) patients (Liu H.Y. et al., 2017; Liu Y.Y. et al., 2017; Liu et al., 2017a,b, 2019). In this study, we aimed to explore whether acupuncture could regulate differentially expressed exosomal miRNAs between patients with MWoA and healthy controls (HCs). Furthermore, we also aimed to identify diagnostic biomarkers that helped differentiate MWoA patients from HCs and identify prognostic biomarkers that helped to predict the effect of acupuncture.

## 2. Materials and methods

### 2.1. Study design, patient recruitment, and ethics approval

We recruited 63 patients who met the MWoA diagnostic criteria of the International Classification of Headache Disorders from the Sichuan Provincial People’s Hospital and the Hospital of Chengdu University of Traditional Chinese Medicine between August 2020 and May 2022 (Headache classification Committee of the International Headache Society (IHS), 2018). They should satisfy the following criteria: (i) aged between 18 and 55 years, with the initial onset of migraines before the age of 50 years; (ii) have experienced two to eight migraine attacks, but fewer than 15 days per month, in the past 3 months and baseline period; (iii) have suffered from the migraine attacks for more than a year; (iv) have completed the headache diary and provided baseline values; (v) have a VAS pain level of 3 to 7 at baseline; (vi) haven not received any acupuncture treatment in the previous 3 months. Individuals who meet any of the following criteria were excluded from the trial: (i) tension-type headache, cluster headache, or other primary headaches; secondary headache disorders; neuralgia of the face or head; (ii) relatively severe systemic diseases (cardiovascular disease, acute infectious diseahepatopathyathy, endocrinopathy, allergy or hyperthyroidism); (iii) severe mental illness, such as severe anxiety and depression; (iv) pregnancy, lactation, or insufficient contraception; (v) involvement in other clinical trials; (vi) inability to read and understand the evaluation scales. Using central randomization, patients with MWoA were randomly assigned to two groups, true acupuncture treatment (trAMWoA) and sham acupuncture treatment (shAMWoA), at a ratio of 1: 1.

We also recruited 32 age- and sex-matched HCs from a community near Chengdu University of Traditional Chinese Medicine between August 2020 and January 2022. This study was approved by the ethics committee of Sichuan Traditional Chinese Medicine, Chengdu, China (permission number 2019KL-030) and was conducted in accordance with the Declaration of Helsinki. The trial was registered in the Chinese Clinical Trial Registry (ChiCTR2000034417). Written informed consent was obtained from all the participants before they were enrolled in the trial.

### 2.2. Interventions

The trAMWoA group and HCs received TA treatment, while the shAMWoA group received SA treatment. Both TA and SA treatments were conducted according to a previously published protocol (Chen et al., 2022) and performed by experienced physicians with more than 5 years of clinical experience in acupuncture treatment. The acupoint prescriptions, acupoint localization, acupoint selection, and insertion depth were described in detail in our previous study (Zhao et al., 2017; Chen et al., 2022). Each needle was inserted and left in place for 30 min. All subjects underwent three sessions per week over a 4-week period. Patients in trAMWoA and shAMWoA groups were asked not to take any prophylactic medications for migraines but were permitted to use ibuprofen (300 mg capsules with sustained release) as a rescue medication if they have severe migraine pain (VAS score > 8) (Dincer and Linde, 2003).

### 2.3. Clinical outcome measures

The frequency of migraine attacks; mean duration of migraine attacks; visual analogue scale (VAS) assessment of pain; time unable to study, work or do housework; migraine-specific QoL questionnaire (MSQ); and Headache Impact Test-6 (HIT-6) scale were used to measure the clinical outcomes of interest in patients with MWoA (Zhao et al., 2017; Xu et al., 2020). VAS scores (0–10, 0 represents painless, 10 represents unbearable excruciating pain) and MSQ scale values were used to measure the pain intensity and the QoL of patients with migraine, respectively (Chang et al., 2019). The MSQ has three scales for assessing QoL: (i) role restriction, which includes seven items that assess the restrictions the MWoA patients experience in daily life due to migraine; (ii) role preventive, which consists of four items that assess how patients’ performance of normal activities is interrupted by migraines; and (iii) emotion function, which consists of three items that assess the impact of migraine on the respondent’s emotions (e.g., frustration or helplessness). The item responses range from one to six (1 = “None of the time,” 2 = “A little bit of time,” 3 = “Some of the time,” 4 = “A good bit of the time,” 5 = “Most of the time,” 6 = “All of the time”). All items were reverse-coded and standardized to a scale of 0–100. Thus, higher scale scores indicate better migraine-related QoL. The HIT-6 scale was designed to assess a broad range of factors that contribute to headache burden and has proven useful in generating quantitative and relevant data on the impact of headaches (Bayliss et al., 2003). The HIT-6 consists of six items: pain, social functioning, role functioning, vitality, cognitive functioning, and psychological distress (Bayliss et al., 2003). The patient responds to each of the six related questions with one of five options: “never,” “rarely,” “sometimes,” “very often,” or “always.” These responses were assigned numeral values added together to yield a total HIT-6 score ranging from 36 to 78, with a higher score indicating a greater impact of headaches on the respondent’s daily life. The clinical outcomes of the TA group were measured during the baseline period and after the 4-week course of TA treatment, and the clinical outcomes of the SA group were measured during the baseline period and after the 4-week course of SA treatment.

### 2.4. Serum collection

We randomly selected 10 subjects from each group and collected the fasting blood samples at the Hospital of Chengdu University of Traditional Chinese Medicine between 8: 00 am and 10: 00 am during the 3-day period immediately before and after the true or sham acupuncture treatment. Notably, for patients in trAMWoA and shAMWoA group, we collected the blood samples during the interictal period (≥24 h headache-free). The serum was extracted by centrifugation at 3,000 rpm for 15 min and immediately stored at −80°C.

### 2.5. Serum exosome isolation and characterization

We used the exoEasy Maxi Kit (product no.: 76064, Qiagen, Hilden, Germany) to isolate and purify the exosomes from serum samples of MWoA patients, according to the manufacturer’s protocols with minor modifications. Briefly, the serum samples were passed through a filter (0.8 μm pore size, product no.: SLAA033SB; Millipore, Burlington, Massachusetts, United States) to exclude large particles. An equivalent volume of kit buffer XBP was then added to 500 μl of the sample, followed by five inversions. The mixture was then added to an exoEasy spin column and centrifuged at 500 *g* for 1 min at room temperature. After the flow-through was discarded, 10 ml of buffer XWP was added to the column and centrifuged at 5,000 *× g* for 5 min to remove residual buffer in the column. The column was then transferred to a fresh collection tube, 400 μl of buffer XE was added, and the mixture was incubated for 1 min. The collection tube was centrifuged at 500 *g* for 5 min to collect the eluate. We used transmission electron microscopy (TEM; FEI Tecnai Spirit T12; FEI, Hillsboro, Oregon, United States) and exosome size analysis (NanoFCM; Xiamen, China) to visualize and analyze the size range of serum exosomes in MWoA patients, respectively, as described previously (Chen et al., 2019). Exosomal markers such as CD9 and TSG101 were characterized by western blotting (WB) as described previously (Ji et al., 2014).

### 2.6. miRNA profiling of exosomes by RNA sequencing

Total RNA was extracted from exosomes using the miRNeasy Serum/Plasma kit (product no. 217184; Qiagen), according to the manufacturer’s protocol. RNA purity and concentration were evaluated using a NanoPhotometer spectrophotometer (Implen, München, Germany) and a Qubit 2.0 fluorometer (Life Technologies, Carlsbad, California, United States), respectively. RNA integrity was assessed using the RNA Nano 6,000 Assay Kit of the Agilent Bioanalyzer 2,100 system (Agilent Technologies, Santa Clara, California, United States). RNA samples (2.5 ng) were used for small RNA sequencing library construction with the NEB Next Ultra small RNA Sample Library Prep Kit (New England Biolabs, Ipswich, Massachusetts, United States), and the libraries were sequenced on the Illumina NovaSeq 6,000 platform (Illumina, San Diego, California, United States). The sequencing data in our manuscript have been deposited in the Bioproject database in NCBI, and the BioProject ID is PRJNA878604.

### 2.7. Analysis of miRNA sequencing data

Raw data were processed using in-house Perl scripts to remove adapters, low-quality reads, and reads containing adapters or poly-N sequences. Clean reads (18–30 nt) were then aligned to the human reference genome (GRCH38) using SOAP2 without mismatches (Li et al., 2009). Then, miRNAs were identified and profiled by mapping the clean reads to human miRNA precursors from miRBase (v22.1). Other small RNAs, such as rRNA, tRNA, snRNA, snoRNA, srcRNA, srpRNA, mRNA fragments, and repeat-associated small RNAs, were identified as described previously (Chen et al., 2019). Before we identified differentially expressed miRNAs (DEmiRs), miRNA expression was normalized using the transcripts per million reads (TPM) method with the total number of clean reads as background. edgeR was used to identify DEmiRs with the following cut-offs: TPM > 5, log2 fold change (log2FC) > 1 or < −1, value of *p* <0.05, and false discovery rate (FDR) < 0.05, as described previously (Chen et al., 2019).

### 2.8. Target gene prediction and functional analysis

We predicted the target genes of selected miRNAs using the Osteo-miRNA-Target-Prediction-Tool,^1^ an easy-to-use tool comprising TargetScan^2^ and miRDB^3^ results for human miRNAs. DAVID Bioinformatics Resources^4^ were used to analyze the potential functions (Kyoto Encyclopedia of Genes and Genomes [KEGG] pathway and Gene Ontology [GO]) of the miRNA target genes.

### 2.9. miRNA validation with a real-time quantitative polymerase chain reaction

Real-time quantitative polymerase chain reaction (RT-qPCR) was performed to confirm the changes in the expression of the candidate miRNAs. U6 small nuclear RNA was used as the internal control. The detailed process of RT-qPCR for hsa-mir-145-5p, hsa-miR-4,732-5p, and hsa-miR-550a-3-5p was described in a previous study (Busk, 2014). The mir-369-5p primer was predicted as described previously and synthesized in the Huayu gene (Guangzhou, China). Total RNA (20 ng) was reverse transcribed using a cDNA synthesis kit (1,708,890; Bio-Rad, Hercules, California, United States). RT-qPCR was performed using SYBR Green qPCR Mix (product no. 1725124; Bio-Rad) on a CFX Connect detection system (Bio-Rad). After the Ct values were detected, ∆Ct was calculated to determine the expression of a miRNA in one sample. ∆∆Ct was used to show the difference in a gene in HC, shAMWoA, and trAMWoA. Reactions were performed in triplicate for each miRNA in each sample. Relative normalized expression (RNE) was used to show the change in gene expression: RNE = 2^–∆∆Ct^. All the primers used are listed in Supplementary Table 1.

### 2.10. Statistical analysis

Statistical analyses were performed using SPSS (version 26.0; IBM, Armonk, New York, United States) and Prism 9.0 (GraphPad, San Diego, California, United States). Two-tailed Student’s t-test was used for between-group comparisons of normally distributed continuous data, and Mann–Whitney *U*-test was used for between-group comparisons of non-normally distributed continuous data. The Wilcoxon matched-pairs signed-rank test was used to compare the non-normally distributed clinical outcome data recorded in trAMWoA and shAMWoA before and after 4-week treatment. The paired t-test was used to compare the normally distributed clinical outcome data recorded in trAMWoA and shAMWoA before and after 4-week treatment. If the baseline characteristics were normally distributed, we planned to use the analysis of covariance to detect differences among HC, trAMWoA, and shAMWoA groups; if not, we planned to use the Kruskal-Wallis test. Statistical significance was set at *p* < 0.05. We then used the AUCRF package to evaluate the importance of each miRNA in distinguishing migraine patients from HCs and to evaluate the prognostic performance of acupuncture treatment in migraine patients by generating 20,000 decision trees, as described previously (Wang et al., 2022). Next, the selected variables were used as a combination to establish a random forest (RF) model by generating 20,000 decision trees using the random Forest package, and the RF model was used to predict the diagnostic and prognostic power of selected miRNAs in patients with migraine. Pearson’s correlation analysis was used to evaluate the correlation between miRNAs and clinical phenotypes.

## 3. Results

### 3.1. Study cohort and migraine treatment using acupuncture

**Figure 1:**
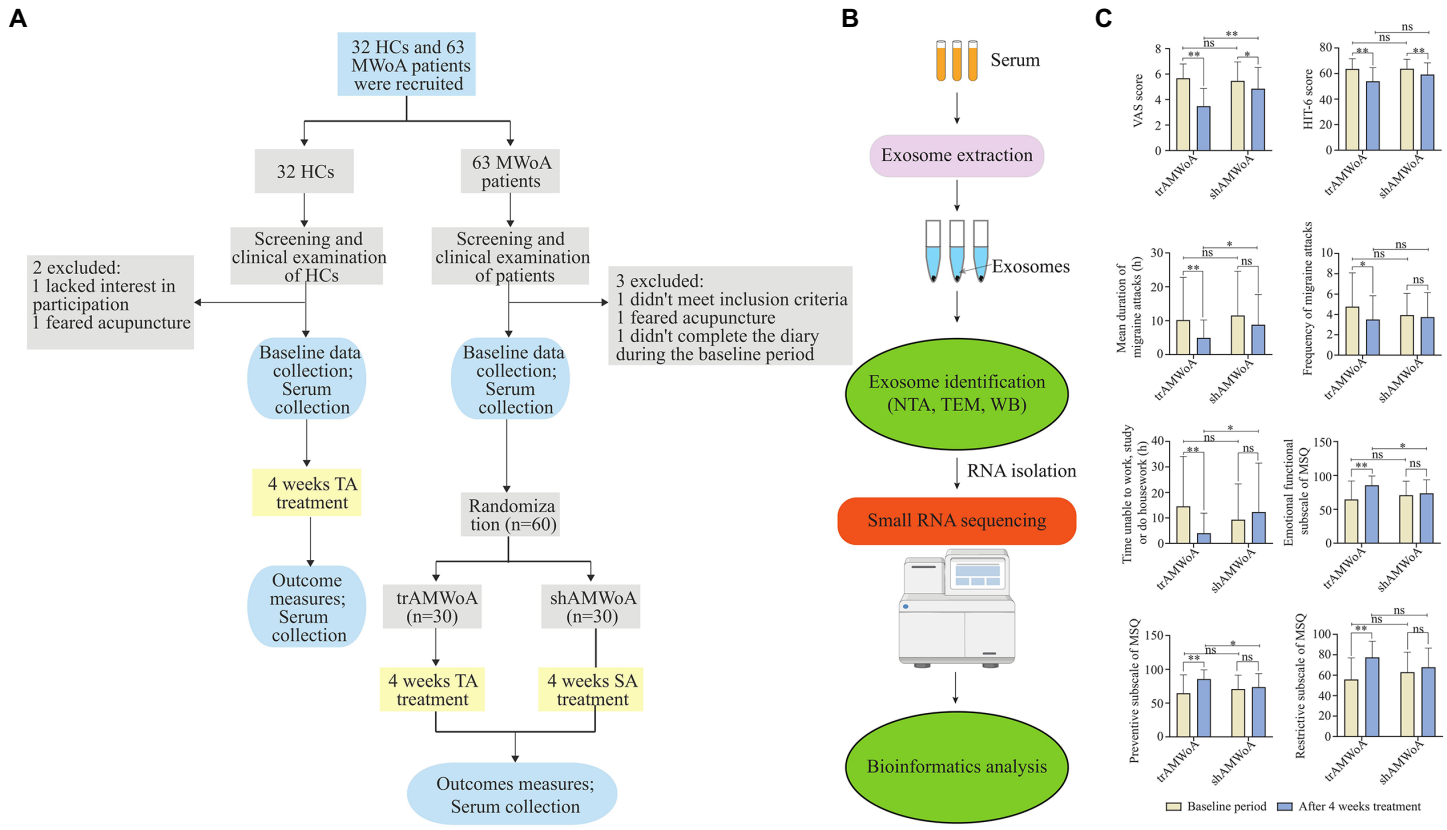
Study outline, sample workflow and clinical outcomes before and after 4-week treatment. **(A)** The detailed process of screening, enrollment, randomization, and treatment. **(B)** Workflow from sample collection to exosome identification, small RNA sequencing and bioinformatics analysis. **(C)** Clinical outcomes of trAMWoA and shAMWoA in baseline and after 4-week treatment. * represents *p* < 0.05, ** represents *p* < 0.01. HCs, healthy controls; trAMWoA, migraine without aura patients receiving true acupuncture treatment; shAMWoA, migraine without aura patients receiving sham acupuncture treatment; TA, true acupuncture; SA, sham acupuncture; NTA, nanoparticle tracking analysis; TEM, transmission electron microscopy; WB, western blotting; VAS, visual analogue scale; Headache Impact Test-6 scale, HIT-6; MSQ, migraine-specific quality of life questionnaire; MWoA, migraine without aura.

The recruitment and exclusion of participants for this study can be seen in Figure 1A and the overall experimental design can be seen in Figure 1B. To study the serum exosomal miRNA signatures for MWoA patients, we recruited 63 MWoA patients who have suffered from migraine attacks for more than 1 year and excluded 3 MWoA patients. During the baseline period, 2 to 6 migraine attacks occurred, and a VAS pain score of 3 to 7 was evaluated for the patients. Patients were randomly assigned to either trAMWoA (*n* = 30) or shAMWoA (*n* = 30) groups using central randomization. No statistically significant differences were observed between the trAMWoA and shAMWoA groups in the headache profile, HIT-6 scores, MSQ scale scores, and migraine symptoms (Table 1). In addition, we recruited 32 age- and sex-matched HCs for this study and excluded 2 of them (Figure 1A). The demographic and clinical characteristics of the study participants are presented in Table 1.

**Table 1:** Demographic and clinical characteristics of study participants (*M*[Q25，Q75]).

	**HC (*n* = 30)**	**trAMWoA (*n* = 30)**	**shAMWoA (*n* = 30)**	***Z*/*H* value**	***p* value**
**Gender (male/female)**	11/19	7/23	7/23	–	–
Age (Y)	32 (24, 47.25)	36 (29.75, 47.25)	36 (27.75, 42)	0.946	0.623
Body weight (Kg)	55.5 (51, 71.25)	59 (50.75, 64.75)	54 (48.75, 65)	1.611	0.447
Systolic pressure (mmHg)	115 (110, 120)	117 (107.8, 120)	114.5 (107.8, 121.5)	0.062	0.97
Diastolic pressure (mmHg)	77 (71.5, 80)	75.5 (70, 80)	72.5 (64.75, 81)	2.143	0.342
Resting heart rate (bpm/ min)	70 (68, 76)	74.5 (65, 83)	72.5 (66.5, 78)	0.765	0.682
***Headache profile***					
Duration of migraine history, mo	–	96 (36, 126)	79 (46.5, 123)	−0.229	0.818
Mean migraine attack durations	–	5.5 (2.75, 12.43)	7.6 (2.83, 12)	−0.599	0.549
Frequency	–	3.5 (3, 5)	3 (3, 4.25)	−0.878	0.38
Intensity, VAS (0–10)	–	5.55 (5, 6.53)	5.59 (4, 6.63)	−0.72	0.472
Familiality, yes/no	–	7/23	7/23	–	–
HIT-6 scores	–	65 (57.8, 68)	65 (59.8, 67.2)	−0.015	0.988
MSQ restrictive subscale scores	–	58.6 (45.7, 71.4)	61.4 (50.7, 71.4)	−0.97	0.332
MSQ preventive subscale scores	–	72.5 (50, 85)	72.5 (60, 81.25)	−0.349	0.727
MSQ emotional functional subscale scores.	–	76.7 (66.7, 88.3)	80 (66.7, 86.7)	−0.247	0.805
Migrainous symptoms, no. (%)	–				
Unilateral	–	18 (60)	22 (73.3)	–	–
Nausea or vomiting	–	21 (70)	17 (56.7)	–	–
Photophobia and phonophobia	–	24 (80)	22 (73.3)	–	–

HC, healthy control; trAMWoA, migraine patinets in true acupuncture group; shAMWoA, migraine patients in sham acupuncture group; HIT-6, Headache Impact Test-6; MSQ, Migraine-specific quality of life questionnaire.

Next, the trAMWoA and HC groups received TA treatment, while the shAMWoA group received SA treatment. We assessed the clinical outcomes of patients with MWoA before and after 4-week treatment. Both the TA and SA treatments produced improvements in VAS and HIT-6 scores. TA treatment improved the mean migraine attack duration, frequency of migraine attacks, time unable to study, work or do housework, MSQ restrictive subscale scores, MSQ preventive subscale scores, and MSQ emotional function subscale scores of the patients. Furthermore, except for the HIT-6 scores, MSQ restrictive subscale scores, and frequency of migraine attacks, trAMWoA patients showed significantly better performance in VAS, mean migraine attack durations, time unable to study, work, or do housework, MSQ emotional functional subscale scores, and MSQ preventive subscale scores after 4-week treatment than shAMWoA patients (Figure 1C; Supplementary Table 2). In addition, patients in the trAMWoA and shAMWoA groups did not take any drugs during the 4-week treatment period. Moreover, we randomly selected 10 patients from the trAMWoA and shAMWoA group, respectively, by using a random number table and collected serum samples from these patients. No statistically significant differences were observed between these patients from trAMWoA and shAMWoA groups in demographic and clinical characteristics (Supplementary Table 3).

### 3.2. Characterization of exosomes extracted from serum

**Figure 2:**
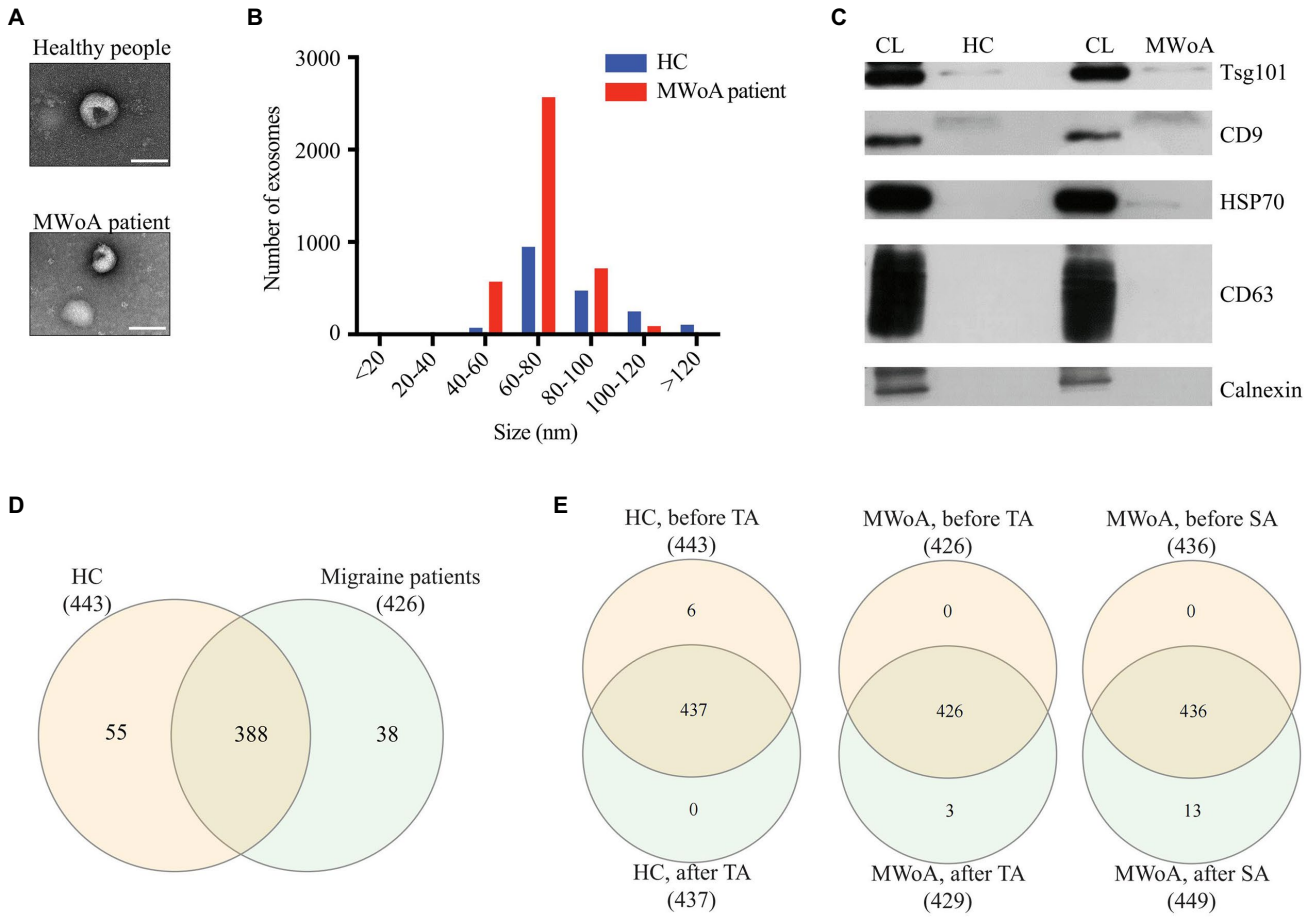
Characterization of exosomes and miRNAs identified in HC, trAMWoA and shAMWoA before and after treatment. **(A)** TEM results of exosomes from MWoA patients and HC. **(B)** The mean size of exosomes of MWoA patients and HC participants by NTA. **(C)** WB revealed CD9 and TSG101 proteins in exosome samples. **(D)** miRNAs identified in HC and MWoA patients. **(E)** miRNAs identified in HC, trAMWoA and shAMWoA before and after 4-week treatment. MWoA, migraine without aura patients; HC, healthy control; TA, true acupuncture; SA, sham acupuncture.

To identify possible serum biomarkers and molecules in response to TA treatment in MWoA patients, we isolated exosomes from the serum of the MWoA and HC groups. TEM results showed that the size of serum exosomes in MWoA patients ranged from 50 to 150 nm (Figure 2A). Further, nanoparticle tracking analysis revealed that the mean size of exosomes of MWoA patients and HC participants was 72.04 nm and 83.57 nm, respectively (Figure 2B). No morphological or particle number differences were observed between MWoA patients and HCs. WB confirmed the presence of exosomal markers CD9 and TSG101 proteins in these samples (Figure 2C). We then performed small RNA sequencing for the exosomes of MWoA patients and HC volunteers before and after acupuncture treatment.

### 3.3. Exosomal miRNA profile of HCs and MWoA patients by small RNA sequencing

Small RNA-seq produced an average of 42 million raw reads, and 27 million reads after low-quality and adapter contamination reads were discarded. We then aligned the clean reads to miRBase (v22.1) and identified 1,154 serum exosomal miRNAs in these samples. After filtering for low expression of miRNAs (TPM < 1), we obtained 561 miRNAs in all samples, of which 443, 426, 437, 429, and 449 were distributed in HCs, MWoA patients before treatment, HCs after TA treatment, trAMWoA after TA treatment, and shAMWoA after SA treatment, respectively (Figures 2D,E). Notably, there were 388 miRNAs consistently detected in both HCs and MWoA patients, 55 miRNAs were specifically detected in HCs, and 38 miRNAs were specifically detected in MWoA patients (Figure 2D). Figure 2E shows that 437, 426, and 436 were detected in the HC, trAMWoA, and shAMWoA cohorts, respectively, before and after TA and SA treatments. Next, we compared the top 10 highly expressed miRNAs in these groups and found that 10 highly expressed miRNAs were almost the same in the HC, trAMWoA, and shAMWoA cohorts before and after TA and SA treatments. Meanwhile, the 10 highly expressed miRNAs were almost the same between HCs and MWoA patients (Supplementary Table 4). We then identified DEmiRs in MWoA patients and HC volunteers before and after acupuncture treatment.

### 3.4. Identification of DEmiRs in MWoA patients and HC volunteers before and after the acupuncture treatment

**Figure 3:**
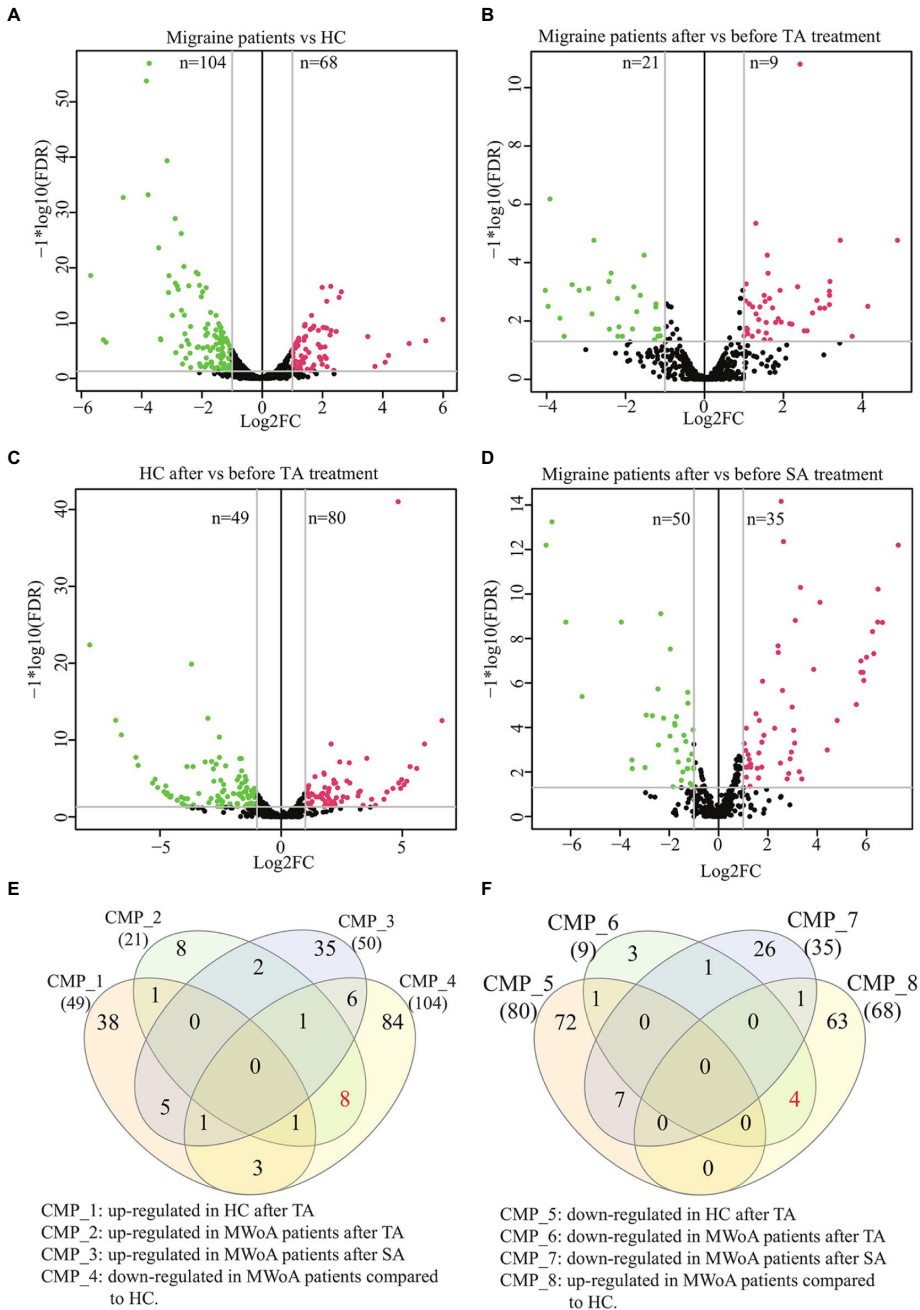
Volcano plots and Venn diagram of DEmiRs in MWoA patients and HC volunteers before and after treatment. **(A)** Volcano plot of DEmiRs between migraine patients and HC. **(B)** Volcano plot of DEmiRs in migraine patients before and after TA treatment. **(C)** Volcano plot of DEmiRs in HC before and after TA treatment. **(D)** Volcano plot of DEmiRs in migraine patients before and after SA treatment. **(E)** Venn diagram of miRNAs up-regulated in HC after TA, up-regulated in MWoA patients after TA, up-regulated in MWoA patients after SA and down-regulated in MWoA patients compared to HC. **(F)** Venn diagram of miRNAs down-regulated in HC after TA, down-regulated in MWoA patients after TA, down-regulated in MWoA patients after SA and up-regulated in MWoA patients compared to HC. MWoA, migraine without aura patients; HC, healthy control; TA, true acupuncture; SA, sham acupuncture; FDR, false discovery rate; log2FC, log2 fold change; CMP, comparison.

A total of 304 DEmiRs were identified in these groups (TMP > 5, log2FC > 1 or < −1, value of *p* <0.05, and FDR < 0.05) (Supplementary Table 5). Compared to HCs, 104 distinct miRNAs were downregulated, whereas 68 distinct miRNAs were upregulated in patients with MWoA (Figure 3A). TA treatment upregulated 49 miRNAs and downregulated 80 miRNAs in HCs, whereas it upregulated 21 miRNAs and downregulated 9 miRNAs in trAMWoA (Figures 3B,C). In addition, SA treatment upregulated 50 miRNAs and downregulated 35 miRNAs in shAMWoA (Figure 3D). Based on the above results, we predicted the target genes of DEmiRs between HCs and MWoA patients and the target genes of DEmiRs in HC, trAMWoA, and shAMWoA groups before and after treatment. KEGG pathway and GO analyses were performed to analyze the potential functions of the miRNA target genes. The DEmiRs between HCs and MWoA patients were mainly enriched in the PI3K-Akt, Hippo, FoxO, and p53 signaling pathways. The target genes of DEmiRs in trAMWoA before and after treatment were mainly enriched in the PI3K-Akt, Hippo, FoxO, and p53 signaling pathways too.

To identify possible exosomal miRNAs that responded to TA treatment in MWoA patients, we plotted a Venn diagram. The Venn diagram demonstrated that TA specifically rescued eight downregulated miRNAs (hsa-miR-4,732-5p, hsa-miR-1,292-5p, hsa-miR-378c, hsa-miR-5,010-3p, hsa-miR-375, hsa-miR-550a-3-5p, hsa-miR-145-5p, and hsa-miR-378a-5p) and four upregulated DEmiRs (hsa-miR-605-3p, hsa-miR-6,862-5p, hsa-miR-369-5p, and hsa-miR-4,654) between HCs and MWoA patients (Figures 3E,F). In summary, we identified 12 exosomal miRNAs that specifically respond to TA treatment in patients with MWoA.

### 3.5. Diagnostic and prognostic biomarker screening

**Figure 4:**
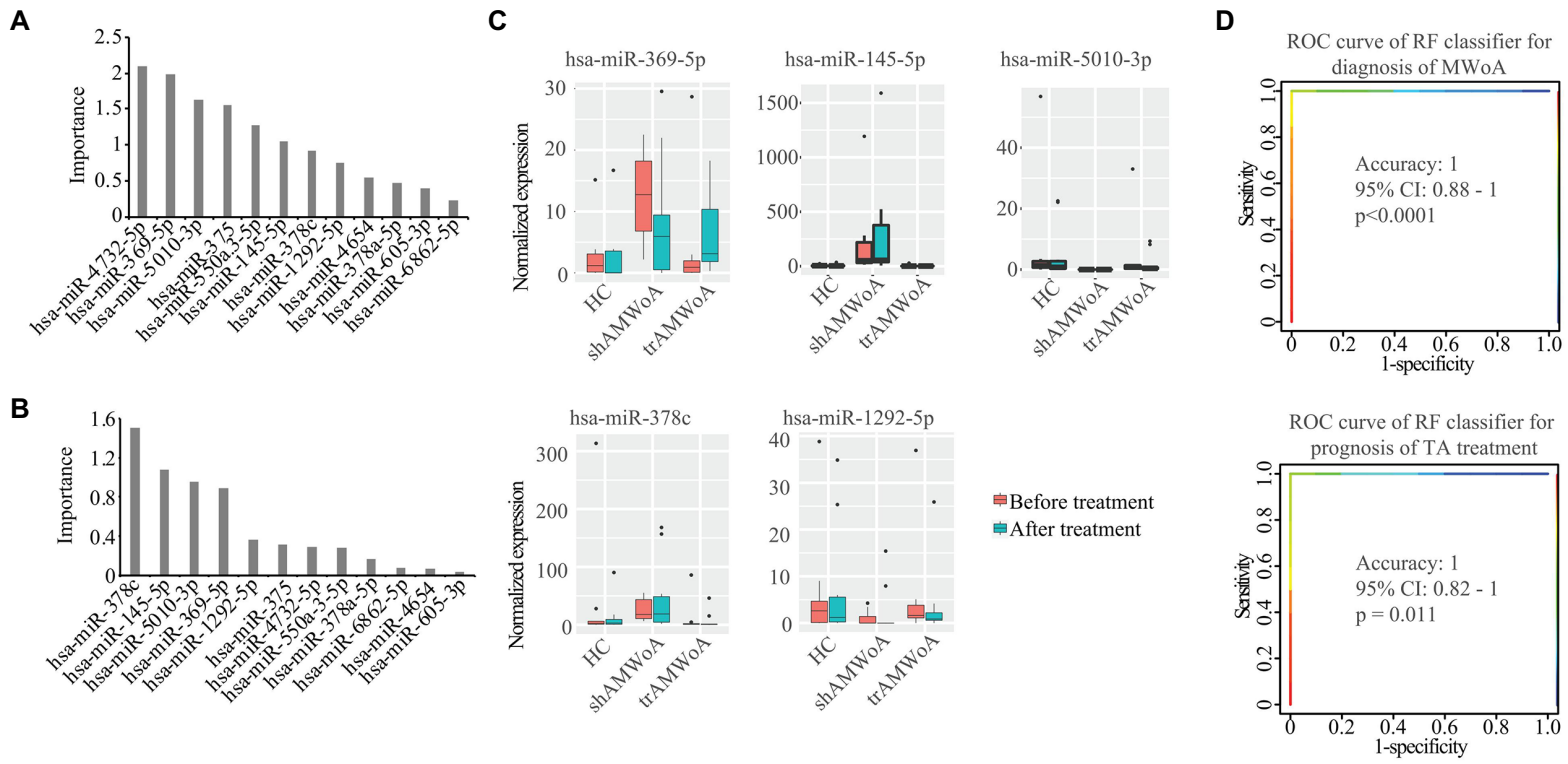
Potential diagnostic and prognostic biomarkers for MWoA patients. **(A)** The importance of these 12 miRNAs in separating MWoA patients and healthy controls. **(B)** The importance of these 12 miRNAs in predicting TA treatment for MWoA patients. **(C)** The changes in expression level of these 5 miRNAs after TA and SA treatment. **(D)** ROC curve of RF classifier for diagnosis of MWoA and ROC curve of RF classifier for prognosis of TA treatment. HC, healthy controls; trAMWoA, migraine without aura patients receiving true acupuncture treatment; shAMWoA, migraine without aura patients receiving sham acupuncture treatment; ROC curve, receiver operating characteristic curve; RF, random forest; TA, true acupuncture; MWoA, migraine without aura patients.

To identify potential diagnostic and prognostic biomarkers for MWoA patients, we first calculated the Pearson correlation between the expression of the 12 exosomal miRNAs and the clinical phenotypes of MWoA patients. There was a significant positive correlation (correlation coefficient = 0.36, *p* = 0.021) between hsa-miR-378a-5p and time unable to work, study, or do housework. Meanwhile, a negative correlation was noted between hsa-miR-605-3p and the restrictive subscale of the MSQ (correlation coefficient = −0.31, *p* = 0.0453). We defined good outcomes as at least a 50% reduction in headache frequency from baseline to postintervention, and poor outcomes as less than a 50% reduction in headache frequency from baseline to post-intervention. There were 14 good-response patients (46.7%) in the trAMWoA group and 5 good-response patients (16.7%) in the shAMWoA group. In patients who donated serum, 6 patients in trAMWoA group were with good outcomes while 4 patients in trAMWoA and 9 patients in shAMWoA were with poor outcomes. We then evaluated the importance of these 12 miRNAs in distinguishing MWoA patients from healthy controls using AUCRF and found that the top six miRNAs were hsa-miR-4,732-5p, hsa-miR-369-5p, hsa-miR-5,010-3p, hsa-miR-375, hsa-miR-550a-3-5p and hsa-miR-145-5p (Figure 4A). We next analyzed the prediction power of these six miRNAs as diagnostic biomarkers for MWoA patients, and the prediction accuracy ranged between 0.83 to 1 (Table 2). When we evaluated the prognostic value of exosomal miRNAs of TA treatment for MWoA patients, the top five miRNAs (hsa-miR-369-5p, hsa-miR-378c, hsa-miR-5,010-3p, hsa-miR-145-5p, and hsa-miR-1,292-5p) showed high importance (Figure 4B) and prediction power (ranged between 0.89 and 1, Table 2). The changes in the expression levels of these five miRNAs after TA and SA treatment are shown in Figure 4C. Notably, three miRNAs, hsa-miR-369-5p, hsa-miR-145-5p, and hsa-miR-5,010-3p were found to have the potential to be diagnostic and prognostic markers. We applied a machine learning method to build a RF model based on these three miRNAs and analyzed the performance of the combination of them. We were not surprised that, based on the combination of these three miRNAs, the machine learning model had high accuracy in predicting MWoA (accuracy: 1; 95% CI: 0.88–1; *p* < 0.0001) and in evaluating TA treatment for MWoA patients (accuracy: 1; 95% CI: 0.82–1; *p* = 0.011) (Figure 4D).

**Table 2:** Prediction power of serum exosomal miRNAs for the diagnosis and prognosis of MWoA patients.

**miRNA**	**Accuracy (95% CI)**	**Value of p**
***Diagnosis***		
hsa-miR-4732-5p	1.00 (0.88–1.00)	0
hsa-miR-369-5p	0.83 (0.65–0.94)	0.0355
hsa-miR-375	0.90 (0.73–0.98)	0.0033
hsa-miR-550a-3-5p	0.90 (0.73–0.98)	0.0033
hsa-miR-145-5p	0.93 (0.78–0.99)	0.0007
hsa-miR-5010-3p	0.93 (0.78–0.99)	0.0007
***Prognosis***		
hsa-miR-369-5p	0.95 (0.74–1.00)	0.068
hsa-miR-145-5p	0.89 (0.67–0.99)	0.2042
hsa-miR-5010-3p	1.00 (0.82–1.00)	0.0112
hsa-miR-378c	0.95 (0.74–1.00)	0.068
hsa-miR-1292-5p	0.95 (0.74–1.00)	0.068

### 3.6. miRNA targeting genes related to migraine

Our sequencing data showed that hsa-let-7b was downregulated in MWoA patients compared with HC volunteers, which was consistent with the results of a previous study (Tafuri et al., 2015). We predicted the target genes of DEmiRs regulated by TA treatment in HCs and patients with MWoA. We found that compared with HCs, hsa-miR-145-5p, hsa-miR-378a-5p, and hsa-miR-550a-3-5p were downregulated, while hsa-miR-369-5p and hsa-miR-605-3p were upregulated in MWoA patients. Notably, TA significantly rescued the expression of these miRNAs. Moreover, hsa-miR-145-5p, hsa-miR-378a-5p, hsa-miR-550a-3-5p, hsa-miR-369-5p, and hsa-miR-605-3p targeted migraine-related genes including *COL4A1*, *NUFIP2*, *TGFBR2*, *MACF1*, *DOCK4*, *PNKD*, and *YAP1* (Gormley et al., 2016; Meng et al., 2018; de Boer et al., 2020; Khan et al., 2021; Table 3). Thus, we inferred that TA might exert a therapeutic effect on migraine by regulating miRNAs that target migraine-related genes.

**Table 3:** miRNAs targeting migraine associated genes.

**Target**	**miRNA**	**MWoA *vs* HC**			**Tramwoa. after *vs* before**			**ShAMWoA. after *vs* before**		
		**Log2FC**	**FDR**	**Regulation**	**Log2FC**	**FDR**	**Regulation**	**Log2FC**	**FDR**	**Regulation**
	hsa-let-7b	−1.016726201	0.0000455	DOWN	−0.030057155	0.88764603	NC	−0.999478111	1	NC
COL4A1, NRP1, NUFIP2	hsa-miR-1,185-1-3p	2.148649927	0.007426955	UP	1.724673511	0.003554682	UP	1.822696492	0.713642388	NC
NUFIP2, TGFBR2	hsa-miR-145-5p	−1.818758747	6.50E-09	DOWN	1.724673511	0.003554682	UP	1.822696492	0.713642388	NC
MACF1	hsa-miR-369-5p	1.674996834	7.61E-10	UP	2.42012439	1.57E-11	DOWN	−0.494265678	0.103533154	NC
TGFBR2	hsa-miR-378a-5p	−1.030745682	0.000307929	DOWN	−1.530297359	5.55E-05	UP	0.689945594	0.005079348	NC
DOCK4	hsa-miR-550a-3-5p	−1.097457089	0.000287425	DOWN	1.647392696	0.001682791	UP	0.094096016	0.982427492	NC
COL4A1, PNKD, TGFBR2, YAP1	hsa-miR-605-3p	1.596526684	0.037217673	UP	1.609698484	0.000230076	DOWN	0	1	NC

HC, healthy control; trAMWoA, migraine without aura patients in true acupuncture group; shAMWoA, migraine without aura patients in sham acupuncture group; FDR, false discovery rate.

### 3.7. Validation of diagnostic and prognostic biomarker

**Figure 5:**
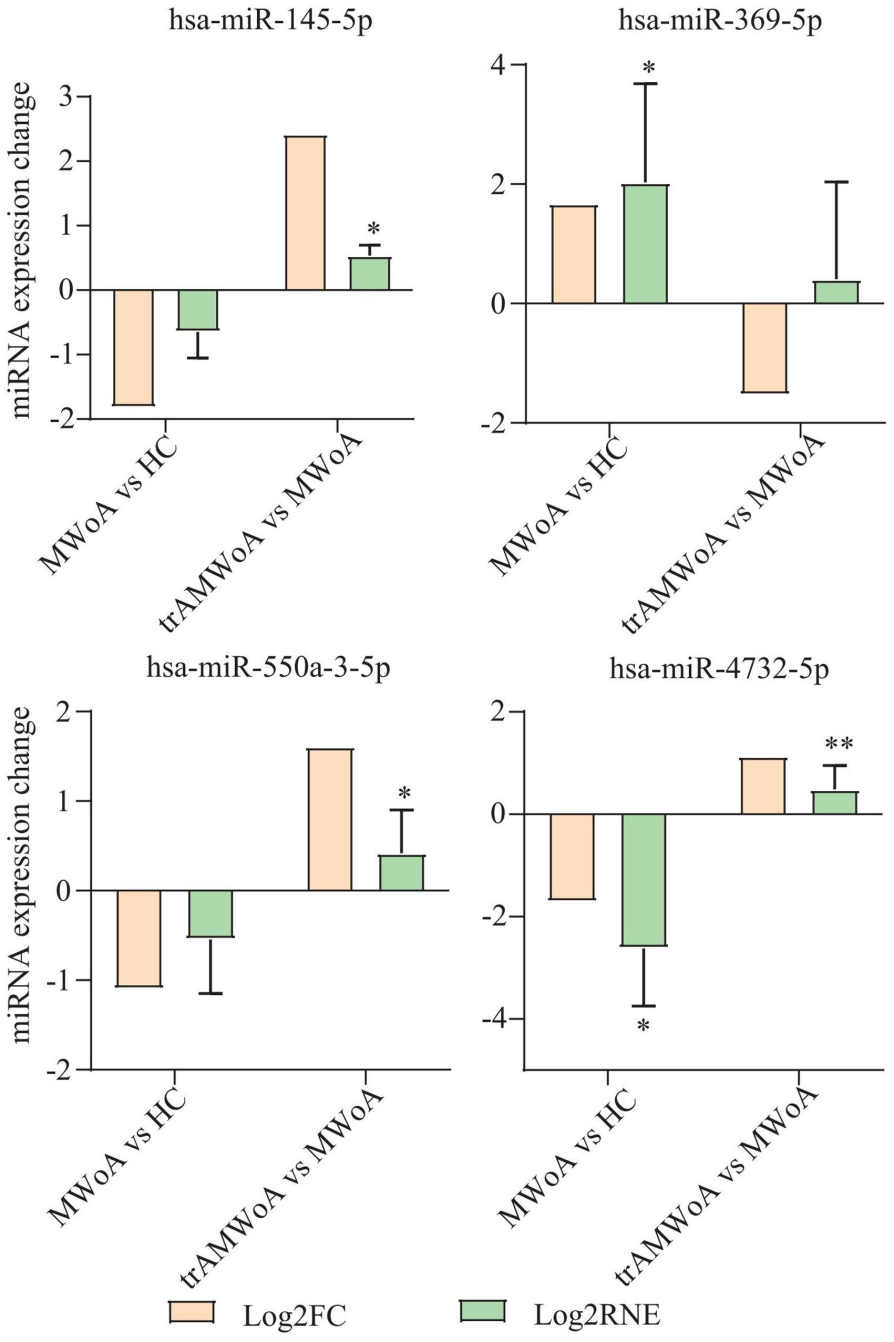
miRNA sequencing results and qPCR results of hsa-miR-369-5p, hsa-miR-4,732-5p, hsa-miR-550a-3-5p, and hsa-mir-145-5p. MWoA, migraine without aura; HC, healthy control; trAMWoA, migraine without aura patients receiving true acupuncture treatment; log2 FC, log2 fold change; log2 RNE, log2 relative normalized expression. * represents *p* < 0.05.

To validate the diagnostic and prognostic biomarkers screened, RT-qPCR was used to examine miRNA expression levels in the serum exosomes of MWoA patients and HC volunteers before and after TA treatment. We selected hsa-miR-369-5p, hsa-miR-4,732-5p, hsa-miR-550a-3-5p, and hsa-mir-145-5p for further validation. The sequencing results illustrated the differential miRNA expression profiles of the four exosomal miRNAs (Figure 5). RT-qPCR showed that hsa-miR-369-5p was upregulated in patients with MWoA relative to HCs and downregulated after TA treatment. Hsa-miR-4,732-5p, hsa-miR-550a-3-5p, and hsa-mir-145-5p were downregulated in MWoA patients relative to the HC group and upregulated after TA treatment. The qPCR results showed the same trend as the miRNA sequencing results (Figure 5).

## 4. Discussion

To the best of our knowledge, this is the first study to identify possible exosomal miRNA targets indicating the efficacy of acupuncture and examine the accuracy of these exosomal miRNAs as diagnostic and prognostic biomarkers. Our study provides a basis for further exploration of diagnostic and prognostic biomarkers for migraine from the perspective of exosomal miRNAs.

In this study, we detected exosomal miRNAs in HC, trAMWoA, and shAMWoA groups before and after the 4-week treatment. The DEmiRs between HCs and MWoA patients were mainly enriched in the PI3K-Akt, Hippo, FoxO, and p53 signaling pathways. The PI3K-Akt signaling pathway is involved in the pathophysiology of migraine (Liu H.Y. et al., 2017; Liu Y.Y. et al., 2017; Liu et al., 2017a,b), and Hippo, FoxO, and p53 signaling pathways are closely related to pain (Li et al., 2013; Chen et al., 2017; Xu et al., 2021). Furthermore, TA exerted a therapeutic effect in MWoA patients, probably by regulating the PI3K-Akt, Hippo, and FoxO signaling pathways. We identified 12 exosomal miRNAs that were specific to TA treatment in patients with MWoA. Among them, hsa-miR-378a-5p was significantly positively related to time unable to work, study, or do housework due to migraine, and hsa-miR-605-3p was significantly negatively related to the restrictive subscale of the MSQ. In a previous study, no miRNA expression level correlated with migraine patients’ headache features, and miR-126 levels were associated with the total number of cardinal migraine symptoms (Cheng et al., 2018). Of note, hsa-miR-605-3p targets *COL4A1*, *PNKD*, *TGFBR2*, and *YAP1*, which have been identified as susceptible genes in migraine patients (Gormley et al., 2016; Khan et al., 2021).

As mentioned above, hsa-miR-369-5p, hsa-miR-145-5p, and hsa-miR-5,010-3p may serve as diagnostic and prognostic markers. Moreover, assessment of the combination of hsa-miR-369-5p, hsa-miR-145-5p, and hsa-miR-5,010-3p could increase the accuracy. It should be noted that miR-369-5p has been reported to target *DNMT3A* (Tao et al., 2018), and overexpression of DNMT3A could significantly lower TRPV1 expression (Bai et al., 2020). TRPV1 plays an important role in the pathophysiology of migraine (Meents et al., 2010). Two studies revealed that miR-145-5p specifically targets Toll-like receptor 4 (TLR4) (Jin et al., 2019; Jiang and Zhang, 2021). Researchers have shown that TLR4 signaling is involved in initiating and maintaining migraine-like behavior and nucleus caudalis neuronal activation in mice (Ramachandran et al., 2019) and that it could promote hyperalgesia by stimulating the production of proinflammatory cytokines and activating microglia in a rat model of migraine (Su et al., 2018). The other three exosomal miRNAs, including hsa-miR-550a-3-5p, hsa-miR-375, and hsa-miR-4,732-5p could be regarded as diagnostic markers. miR-550a-3-5p specifically targeted *YAP1* (Wei et al., 2021), and *YAP1* was one of the migraine susceptibility genes (Gormley et al., 2016). miR-375 is regarded as a diagnostic biomarker in migraine children and adolescents with migraine (Gallelli et al., 2019). However, compared with HCs, the expression level of miR-375 in migraine patients was the opposite in their study compared to ours. A study reported that, unlike whole plasma miRNA, exosomes extracted from the plasma of women with preeclampsia exhibited a unique miRNA profile (Li et al., 2020). Thus, the reasons for the inconsistent results might be that they detected miRNAs in serum, and we detected miRNAs in exosomes; furthermore, they only enrolled children and adolescents, while we enrolled adults between 18 and 55 years old. However, there are relatively few studies on hsa-miR-4,732-5p, which has served as a diagnostic biomarker in epithelial ovarian cancer and breast cancer (Zhang et al., 2015; Liu et al., 2021). Further studies are needed to explore the specific roles of these miRNAs in migraines.

Compared with healthy controls, patients with migraine have upregulated expression of some miRNAs [e.g., miR-155, miR-126, let-7 g, Cheng et al. (2018) miR-34a-5p, and miR-375 (Gallelli et al., 2019)] and downregulated expression of other miRNAs [e.g., miR-30a (Zhai and Zhu, 2018)]. Few studies have investigated the differences in exosomal miRNAs between migraine patients and HCs. Tafuri et al. compared plasma-derived exosomal miRNAs between female patients with MWoA and HCs and found that the expression of miR-27b was upregulated, whereas the expression of miR-181a, let-7b, and miR-22 was downregulated (Tafuri et al., 2015). Notably, our sequence data showed the same trend of let-7b between HCs and MWoA patients as their study. To our knowledge, this is the first study to explore differentially expressed serum-derived exosomal miRNAs between patients with MWoA and HCs. Because of the high accuracy of these miRNAs, our study suggests that these exosomal miRNAs may be used to diagnose migraine, although further large-scale studies are required for confirmation.

We also screened two more miRNAs that could serve as prognostic biomarkers for TA treatment in MWoA patients, namely, hsa-miR-378c and hsa-miR-1,292-5p. In a previous study, miR-378c directly targeted neuropilin 1(NRP1; Hu and Luo, 2022), *NRP1* is also a migraine susceptibility gene (Gormley et al., 2016). However, a small number of studies have explored the function of hsa-miR-1,292-5p and showed it could be used to predict the prognosis of gastric cancer (Xu et al., 2021). Previous findings have highlighted a functional cross-talk between pharmacological treatment and miRNAs, as drug administration could indirectly affect miRNA expression profiles (Rukov et al., 2011), and conversely, some specific miRNAs could be relevant as indicators of drug response (Pehserl et al., 2016). One study suggested that hsa-miR-34a-5p and hsa-miR-375 could be useful biomarkers of disease and drug efficacy in patients with MWoA (Gallelli et al., 2019). Although the number of related studies is quite limited, we know that specific miRNAs could serve both as new molecular targets for drugs and as biomarkers for detecting drug responses and efficacy. Five prognostic markers were identified in our study. These five exosomal miRNAs may be new molecular targets for the alleviation of migraine and could be used to detect acupuncture responses and predict the efficacy of acupuncture.

Gormley et al. (2016) conducted a meta-analysis of 375,000 individuals and identified 38 susceptibility genomic loci for migraine, including *DOCK4*, *NRP1*, *TGFBR2*, and *YAP1*. Other studies have also summarized migraine-related genes, including *COL4A1*, *PNKD*, *MACF1*, and *NUFIP2* (Meng et al., 2018; de Boer et al., 2020; Khan et al., 2021). Among the differentially expressed miRNAs between MWoA patients and HCs, hsa-miR-605-3p, hsa-miR-145-5p, hsa-miR-378a-5p, and hsa-miR-369-5p were rescued by TA treatment. Moreover, hsa-miR-605-3p specifically targets *COL4A1*, *PNKD*, *TGFBR2*, and *YAP1*. *NUFIP2* and *TGFBR2* are targeted by hsa-miR-145-5p. *TGFBR2* is targeted by hsa-miR-378a-5p. *MACF1* is targeted by hsa-miR-369-5p. *DOCK4* is targeted by hsa-miR-550a-3-5p. Thus, we inferred that TA probably exerts a therapeutic effect in treating migraine by regulating exosomal miRNAs that directly target migraine-related genes.

The major limitation of this study is the sample size, which limited the analysis of the effect of gender differences on exosomal miRNA expression in migraine patients. In the future, more samples will be recruited to validate the results in this study and improve the prediction model of migraine patients. Animal studies are needed to determine if the mechanism of action of acupuncture is mediated by exosomal miRNAs. Moreover, the functions and targets of these three miRNAs (hsa-miR-369-5p, hsa-miR-145-5p, and hsa-miR-5,010-3p) need further exploration *in vitro* or *in vivo* in the future.

## 5. Conclusion

We identified three miRNAs that could serve as diagnostic biomarkers to predict the therapeutic effects of TA. Our study provides a basis for further exploration of diagnostic and prognostic biomarkers for migraine from the perspective of exosomal miRNAs. Moreover, we inferred that the underlying mechanism of TA treatment was probably the regulation of exosomal miRNAs that directly target migraine-related genes.

## Data availability statement

The datasets presented in this study can be found in online repositories. The names of the repository/repositories and accession number (s) can be found at: https://www.ncbi.nlm.nih.gov/, PRJNA878604.

## Ethics statement

The studies involving human participants were reviewed and approved by this study was approved by the Ethics Committee of Sichuan Traditional Chinese Medicine (Chengdu, China) in July 2020 and was conducted in accordance with the Declaration of Helsinki. The permission number for the trial was 2019KL-030. Written consent was obtained from all participants before they began participating in the trial. The patients/participants provided their written informed consent to participate in this study.

## Author contributions

LL, WQ, DJC, and LZ designed the study hypotheses and experiments. YW, XN, SG, ZZ, DHC, and ZH developed the data analysis method. LL and WQ were responsible for preparing the tables and figures. All authors contributed to the scientific discussion of the manuscript. All authors participated in the manuscript review and writing.

## Funding

This study was supported by the National Key Research and Development Project (Grant No. 2019YFC1709700), the National Natural Science Foundation of China (Grant Nos. 81973962, 82274664, 82004486, and 82205286), the Department of Science and Technology of Sichuan Province (20ZDYF1199 and 2021ZYD0103), the Innovation Team and Talents Cultivation Program of the National Administration of Traditional Chinese Medicine (Grant No. ZYYCXTD-D-202003), and China Postdoctoral Science Foundation (2020M683643XB). The funding sources had no role in the writing or submission of this article for publication.

## Conflict of interest

The authors declare that the research was conducted in the absence of any commercial or financial relationships that could be construed as a potential conflict of interest.

## Publisher’s note

All claims expressed in this article are solely those of the authors and do not necessarily represent those of their affiliated organizations, or those of the publisher, the editors and the reviewers. Any product that may be evaluated in this article, or claim that may be made by its manufacturer, is not guaranteed or endorsed by the publisher.

## Footnotes

^1^ github.com/WeiYang-BAI/Osteo-miRNA-Target-Prediction-Tool

^2^ http://www.targetscan.org/

^3^ http://mirdb.org

^4^ https://david.ncifcrf.gov

## Abbreviations

miRNA, microRNA
HIT-6, Headache Impact Test-6
MSQ, Migraine-Specific Quality of Life Questionnaire
MWoA, migraine without aura
VAS, visual analog scale
TEM, transmission electron microscopy
GO, gene ontology
KEGG, Kyoto Encyclopedia of Genes and Genomes
RT-qPCR, real-time quantitative polymerase chain reaction
TLR4, Toll-like receptor 4
IFNγ-DC-Exos, interferon gamma–stimulated dendritic cell exosomes
TA, true acupuncture
SA, sham acupuncture
trAMWoA, migraine without aura in the acupuncture group
shAMWoA, migraine without aura in the sham acupuncture group
HC, healthy control
fMRI, functional magnetic resonance imaging
DEmiRs, differentially expressed miRNAs
