# Corrigendum: Circulating exosomal microRNA profiles in migraine patients receiving acupuncture treatment: a placebo-controlled clinical trial

**DOI:** 10.3389/fnmol.2023.1174810

**Published:** 2023-04-20

**Authors:** Lu Liu, Wenchuan Qi, Yanan Wang, Xixiu Ni, Shan Gao, Ziyang Zhou, Daohong Chen, Zhenxi He, Mingsheng Sun, Ziwen Wang, Dingjun Cai, Ling Zhao

**Affiliations:** ^1^Acupuncture and Tuina School, Chengdu University of Traditional Chinese Medicine, Chengdu, Sichuan, China; ^2^Acupuncture and Chronobiology Key Laboratory of Sichuan Province, Chengdu, Sichuan, China

**Keywords:** acupuncture, migraine, biomarker, exosome, miRNA

In the published article, there was an error in Table 2 as published. The prognostic biomarker hsa-miR-550a-3-5p should be corrected to hsa-miR-145-5p, and hsa-miR-4732-5p should be corrected to hsa-miR-5010-3p. The corrected Table 2 and its caption appear below.

**Table 2:** Prediction power of serum exosomal miRNAs for the diagnosis and prognosis of MWoA patients.

**miRNA**	**Accuracy (95% CI)**	***p*-value**
**Diagnosis**		
hsa-miR-4732-5p	1.00 (0.88–1.00)	0
hsa-miR-369-5p	0.83 (0.65–0.94)	0.0355
hsa-miR-375	0.90 (0.73–0.98)	0.0033
hsa-miR-550a-3-5p	0.90 (0.73–0.98)	0.0033
hsa-miR-145-5p	0.93 (0.78–0.99)	0.0007
hsa-miR-5010-3p	0.93 (0.78–0.99)	0.0007
**Prognosis**		
hsa-miR-369-5p	0.95 (0.74–1.00)	0.068
hsa-miR-145-5p	0.89 (0.67–0.99)	0.2042
hsa-miR-5010-3p	1.00 (0.82–1.00)	0.0112
hsa-miR-378c	0.95 (0.74–1.00)	0.068
hsa-miR-1292-5p	0.95 (0.74–1.00)	0.068

In the published article, there was an error in Supplementary Table 1. We mistakenly used “has-miR” instead of “hsa-miR.” Therefore, we corrected Has-miR-145-5p-R and Has-miR-145-5p-F to Hsa-miR-145-5p-R and Hsa-miR-145-5p-F. The correct material statement has been updated in the original article.

In the published article, there was an error. A correction has been made to Section 3.3 **Exosomal miRNA profile of HCs and MWoA patients by small RNA sequencing**. The number of miRNAs consistently detected in both healthy controls and MWoA patients in section 3.3 is 388, not 338 as mentioned in the article.

The corrected sentence appears below:

“[Notably, there were 388 miRNAs consistently detected in both HCs and MWoA patients]”.

In the published article, there was an error. We mistakenly used “has-miR” instead of “hsa-miR” in our article. Therefore, we corrected all instances of “has-mir” to “hsa-mir” in the manuscript.

The authors apologize for these errors and state that they do not change the scientific conclusions of the article in any way. The original article has been updated.

