# Supplementary material for: Circulating exosomal microRNA profiles in migraine patients receiving acupuncture treatment: A placebo-controlled clinical trial
Source: Front Mol Neurosci. 2023 Jan 10;15:1098766. doi: 10.3389/fnmol.2022.1098766 (PMC9871901; doi:10.3389/fnmol.2022.1098766)
Supplement: Supplementary file 1 [file Table_1.DOCX]

Supplementary Materials

# Supplementary Tables

## Supplementary Table 1. Primer information

| **Primer name** | **The sequence**（5’ to 3’） |
| --- | --- |
| U6-F | CTCGCTTCGGCAGCACA |
| U6-R | AACGCTTCACGAATTTGCGT |
| Hu-miR-369-5p-RT | CTCAACTGGTGTCGTGGAGTCGGCAATTCAGTTGAGGCGAAT |
| Hu-miR-369-5p-F | ACACTCCAGCTGGGAGATCGACCGTGTTAT |
| Hsa-miR-4732-5p-R | GTCCAGTTTTTTTTTTTTTTTAGCTTC |
| Hsa-miR-4732-5p-F | GCAGTGTAGAGCAGGGAGCAG |
| Hsa-miR-550a-3-5p-R | GTCCAGTTTTTTTTTTTTTTTCTCTT |
| Hsa-miR-550a-3-5p-F | GCAGAGTGCCTGAGGGAGT |
| Hsa-miR-145-5p-R | TCCAGTTTTTTTTTTTTTTTAGGGATTC |
| Hsa-miR-145-5p-F | GCAGGTCCAGTTTTCCCA |

##

## 1.2 Supplementary Table 2. The comparison of clinical outcomes before and after TA and SA, and the comparison of clinical outcomes between the trAMWoA group and the shAMWoA group after 4 weeks of treatment (M[Q25, Q75]).

| **Clinical outcomes** | | **Baseline period** | **After 4 weeks TA treatment** | | **Z value** | **P value** |
| --- | --- | --- | --- | --- | --- | --- |
| Frequency of migraine attacks(time) | | 3.50 (3.00, 5.00) | 3.00 (2.00, 5.00) | | -2.287 | 0.022 |
| Mean migraine attack duration(h) | | 5.50 (2.75, 12.43) | 3.40 (2.00, 5.25) | | -3.485 | 0.000 |
| time unable to study, work or do housework(h) | | 4.00 (0.375, 26.00) | 0.00 (0.00, 4.50) | | -3.427 | 0.001 |
| VAS score | | 5.55 (5.00, 6.53) | 4.00 (3.00, 4.45) | | -4.713 | 0.000 |
| HIT-6 score | | 65.00 (57.75, 68.00) | 56.5 (42.00, 63.00) | | -4.317 | 0.000 |
| MSQ restrictive subscale score | | 58.57 (45.71, 71.43) | 75.72 (60.00, 90.00) | | -4.42 | 0.000 |
| MSQ preventive subscale score | | 72.5 (50.00, 85.00) | 87.5 (75, 100) | | -4.171 | 0.000 |
| MSQ emotional functional subscale score | | 76.67 (66.67, 88.34) | 93.33(73.33, 100) | | -3.295 | 0.001 |
| **Clinical outcomes** | **Baseline period** | | **After 4 weeks SA treatment** | **Z value** | | **P value** |
| Frequency of migraine attacks(time) | 3.00 (3.00, 4.25) | | 3.00 (2.00, 4.25) | -0.916 | | 0.36 |
| Mean migraine attack duration(h) | 7.59 (2.83, 12.00) | | 6.25 (2.75, 10.5) | -1.756 | | 0.079 |
| time unable to study, work or do housework(h) | 4.50 (0.00, 12.25) | | 5.00 (0.00, 15.75) | -0.578 | | 0.563 |
| VAS score | 5.59(4.00, 6.63) | | 5.00(4.00, 6.06) | -2.24 | | 0.025 |
| HIT-6 score | 65.00 (59.75, 67.25) | | 61.5 (53.00, 65.25) | -2.876 | | 0.004 |
| MSQ restrictive subscale score | 61.43 (50.72, 71.43) | | 71.43(50.72, 83.57) | -1.674 | | 0.094 |
| MSQ preventive subscale score | 72.50 (60.00,81.25) | | 77.50(60.00, 95.00) | -1.104 | | 0.27 |
| MSQ emotional functional subscale score | 80.00 (66.67, 86.67) | | 83.34(66.67, 93.33) | -0.568 | | 0.57 |
| **Clinical outcomes** | | **trAMWoA group** | **shAMWoA group** | | **Z value** | **P value** |
| Frequency of migraine attacks(time) | | 3.00 (2.00, 5.00) | 3.00 (2.00, 4.00) | | -0.067 | 0.946 |
| Mean migraine attack duration(h) | | 3.40 (2.00, 5.25) | 6.25 (2.75, 10.5) | | -2.144 | 0.032 |
| time unable to study, work or do housework(h) | | 0.00 (0.00, 4.50) | 5.00 (0.00, 15.75) | | -2.574 | 0.010 |
| VAS score | | 4.00 (3.00, 4.45) | 5.00 (4.00, 6.06) | | -3.539 | 0.000 |
| HIT-6 score | | 56.5 (42.00, 63.00) | 61.5(53.00, 65.25) | | -1.711 | 0.087 |
| MSQ restrictive subscale score | | 75.72 (60.00, 90.00) | 71.43 (50.72, 83.57) | | -1.795 | 0.073 |
| MSQ preventive subscale score | | 87.50 (75.00, 100) | 77.5(60.00, 95.00) | | -2.092 | 0.036 |
| MSQ emotional functional subscale score | | 93.33 (73.33, 100) | 83.34 (66.67, 93.33) | | -2.051 | 0.040 |

**Note:** Non-normally distributed clinical outcome data, thus the Wilcoxon matched-pairs signed-rank test was used to compare the baseline period data with after-treatment data; Non-normally distributed clinical outcome data, thus the Mann-Whitney U-test was used for comparisons between trAMWoA group and shAMWoA group, statistical significance was set at P < 0.05; **Abbreviations:** M, [Q25, Q75], median [lower quartile, upper quartile]; trAMWoA, migraine without aura patients after true acupuncture treatment; shAMWoA, migraine without aura patients after sham acupuncture treatment; VAS, visual analog scale; HIT-6, headache impact test-6; MSQ, migraine-specific quality of life questionnaire; TA, true acupuncture; SA, sham acupuncture

**1.3 Supplementary Table 3. Demographic and clinical characteristics of 10 patients selected from trAMWoA and shAMWoA group(M[Q25, Q75]).**

|  | **trAMWoA (n=10)** | **shAMWoA (n=10)** | **Z value** | **P value** |
| --- | --- | --- | --- | --- |
| **Gender (male/female)** | 4/6 | 4/6 | - | - |
| **Age (Y)** | 38 (31.25, 47.5) | 36 (28, 40) | -0.908 | 0.364 |
| **Body weight (Kg)** | 60 (54, 72) | 60.3 (51.8, 70.5) | -0.038 | 0.97 |
| **Systolic pressure (mmHg)** | 118 (114, 128.8) | 114 (107.8, 120.3) | -0.721 | 0.471 |
| **Diastolic pressure (mmHg)** | 75 (70, 81.3) | 72.5 (67.3, 82) | -0.53 | 0.596 |
| **Resting heart rate(bpm/ min)** | 66 (61, 77) | 74 (65, 83) | -1.402 | 0.161 |
| **Headache profile** |  |  |  |  |
| Duration of migraine history, mo | 108.5 (54, 159) | 51 (27, 102.5) | -1.438 | 0.15 |
| Mean migraine attack durations | 5.75 (1.75, 19.88) | 6.15 (2, 15) | -0.152 | 0.879 |
| Frequency | 4 (2.75, 6) | 3 (3, 3.25) | -1.074 | 0.283 |
| Intensity, VAS (0–10) | 5.7 (5, 7) | 5.5 (4, 7) | -0.7 | 0.484 |
| Familiality, yes/no | 2/8 | 1/9 | - | - |
| **HIT-6 scores** | 63 (55.5, 66.5) | 65 (64.8, 67.3) | -1.145 | 0.252 |
| **MSQ restrictive subscale scores** | 70 (48.6, 77.9) | 58.6 (50, 67.1) | -1.253 | 0.21 |
| **MSQ preventive subscale scores** | 82.5 (50, 95) | 67.5 (60, 81.3) | -0.802 | 0.423 |
| **MSQ emotional functional subscale scores.** | 83.3 (65, 90) | 73.3 (66.7, 81.7) | -0.924 | 0.356 |
| **Migrainous symptoms, no. (%)** |  |  |  |  |
| Unilateral | 4 (40) | 8 (80) | - | - |
| Nausea or vomiting | 5 (50) | 6 (60) | - | - |
| Photophobia and phonophobia | 7 (70) | 8 (80) | - | - |

Note: Non-normally distributed clinical outcome data, thus the Mann-Whitney U-test was used for comparisons between trAMWoA group and shAMWoA group, statistical significance was set at P < 0.05; **Abbreviations:** M, [Q25, Q75], median [lower quartile, upper quartile]; trAMWoA, migraine patients in true acupuncture group; shAMWoA, migraine patients in sham acupuncture group; VAS, visual analog scale; HIT-6, headache impact test-6; MSQ, migraine-specific quality of life questionnaire.

## 1.4 Supplementary Table 4. The top 10 highly expressed miRNAs in MWoA patients and HC before and after treatment.

| **miRNA** | **TPM** | |
| --- | --- | --- |
| **HC before TA** | | |
| hsa-miR-486-5p | | 37102.4218 |
| hsa-miR-451a | | 35212.4221 |
| hsa-miR-122-5p | | 31791.7796 |
| hsa-let-7b-5p | | 13356.1796 |
| hsa-miR-126-3p | | 11945.9046 |
| hsa-let-7a-5p | | 10073.07 |
| hsa-miR-423-5p | | 9889.3841 |
| hsa-miR-92a-3p | | 9811.1496 |
| hsa-let-7i-5p | | 8669.2693 |
| hsa-miR-26a-5p | | 7862.7834 |
| **HC after TA** | | |
| hsa-miR-486-5p | 35806.7338 | |
| hsa-miR-451a | 34912.1621 | |
| hsa-miR-122-5p | 30250.7031 | |
| hsa-let-7b-5p | 11688.1215 | |
| hsa-miR-126-3p | 10448.6836 | |
| hsa-miR-92a-3p | 9544.7117 | |
| hsa-miR-423-5p | 9512.9054 | |
| hsa-let-7a-5p | 8896.5034 | |
| hsa-let-7i-5p | 8164.4991 | |
| hsa-miR-25-3p | 7441.7909 | |
| **MWoA patients** | | |
| hsa-miR-451a | 42928.2569 | |
| hsa-miR-122-5p | 40666.0826 | |
| hsa-miR-486-5p | 30479.9425 | |
| hsa-let-7b-5p | 17263.129 | |
| hsa-let-7i-5p | 14735.7018 | |
| hsa-miR-26a-5p | 11882.3863 | |
| hsa-miR-126-3p | 10625.1069 | |
| hsa-miR-423-5p | 10449.541 | |
| hsa-miR-148a-3p | 10423.5994 | |
| hsa-let-7a-5p | 9493.58 | |
| hsa-miR-451a | 42928.2569 | |
| **TrAMWoA after TA treatment** | | |
| hsa-miR-451a | 42959.4577 | |
| hsa-miR-122-5p | 40777.7469 | |
| hsa-miR-486-5p | 30575.5639 | |
| hsa-let-7b-5p | 17535.9393 | |
| hsa-let-7i-5p | 15318.3217 | |
| hsa-miR-26a-5p | 12523.5885 | |
| hsa-miR-126-3p | 10967.4898 | |
| hsa-miR-423-5p | 10701.8425 | |
| hsa-miR-148a-3p | 10572.9394 | |
| hsa-let-7a-5p | 10032.1886 | |
| **ShAMWoA after SA treatment** | | |
| hsa-miR-148a-3p | 53054.8117 | |
| hsa-miR-151a-3p | 49777.791 | |
| hsa-miR-122-5p | 47171.0264 | |
| hsa-let-7i-5p | 25185.5687 | |
| hsa-miR-21-5p | 24516.5242 | |
| hsa-miR-126-3p | 20483.5602 | |
| hsa-miR-26a-5p | 19061.0112 | |
| hsa-miR-451a | 12666.0995 | |
| hsa-miR-143-3p | 11231.4952 | |
| hsa-let-7f-5p | 10434.3021 | |

Notes: HC, healthy control; trAMWoA, migraine patients in true acupuncture group; shAMWoA, migraine patients in sham acupuncture group; TA, true acupuncture; SA, sham acupuncture; TPM, transcripts per million reads.

## 1.5 Supplementary Table 5. DEmiRs in MWoA patients and HC volunteers before and after the 4-week treatment

| **miRNA** | | **Log2FC** | | **FDR** | | **Regulation** |
| --- | --- | --- | --- | --- | --- | --- |
| **DEmiRs between MWoA patients and HC volunteers** | | | | | | |
| hsa-miR-1296-5p | | 1.365804648 | | 0.001427128 | | UP |
| hsa-miR-885-5p | | -1.410374312 | | 7.99E-08 | | DOWN |
| hsa-miR-410-3p | | 1.129470281 | | 0.021597015 | | UP |
| hsa-miR-193b-3p | | -2.394906474 | | 2.66E-05 | | DOWN |
| hsa-miR-939-5p | | -1.830282697 | | 0.002789468 | | DOWN |
| hsa-miR-517a-3p | | -2.045136273 | | 0.006618568 | | DOWN |
| hsa-miR-147b | | -1.68526724 | | 0.001046139 | | DOWN |
| hsa-miR-6815-5p | | -2.434906929 | | 6.49E-07 | | DOWN |
| hsa-miR-133a-3p | | -2.688058741 | | 6.43E-27 | | DOWN |
| hsa-miR-195-5p | | -1.31185653 | | 3.53E-05 | | DOWN |
| hsa-miR-483-3p | | -1.513780452 | | 3.84E-08 | | DOWN |
| hsa-miR-4732-5p | | -1.714594845 | | 1.18E-12 | | DOWN |
| hsa-miR-200a-3p | | -2.190422201 | | 6.70E-20 | | DOWN |
| hsa-miR-381-3p | | 1.011631761 | | 2.45E-05 | | UP |
| hsa-miR-548f-3p | | -1.578532645 | | 0.03883803 | | DOWN |
| hsa-miR-1226-5p | | -2.682881923 | | 1.46E-06 | | DOWN |
| hsa-miR-2355-3p | | 1.443718184 | | 2.50E-08 | | UP |
| hsa-miR-365b-3p | | -1.871115839 | | 1.75E-08 | | DOWN |
| hsa-miR-136-5p | | -1.00570002 | | 0.000104989 | | DOWN |
| hsa-miR-499a-5p | | -1.581639259 | | 1.75E-08 | | DOWN |
| hsa-miR-6859-5p | | -1.011632308 | | 0.036232007 | | DOWN |
| hsa-miR-6852-5p | | 1.443017046 | | 5.26E-06 | | UP |
| hsa-miR-3605-3p | | -1.115337855 | | 0.000191835 | | DOWN |
| hsa-miR-1224-5p | | -1.061427538 | | 0.005532134 | | DOWN |
| hsa-miR-25-5p | | -1.335048383 | | 4.06E-07 | | DOWN |
| hsa-miR-149-5p | | -1.37338539 | | 0.001844203 | | DOWN |
| hsa-miR-132-3p | | -1.264988682 | | 0.002254452 | | DOWN |
| hsa-miR-340-5p | | 1.123795964 | | 1.09E-06 | | UP |
| hsa-let-7f-5p | | 1.205683157 | | 5.47E-08 | | UP |
| hsa-miR-1185-1-3p | | 2.148649927 | | 0.007426955 | | UP |
| hsa-miR-1292-5p | | -1.020714494 | | 0.002302328 | | DOWN |
| hsa-miR-4662a-5p | | 2.45291004 | | 3.19E-09 | | UP |
| hsa-miR-365a-5p | | -2.566167168 | | 8.53E-09 | | DOWN |
| hsa-miR-148b-3p | | 1.065059087 | | 1.87E-06 | | UP |
| hsa-miR-625-3p | | 1.057567365 | | 3.42E-06 | | UP |
| hsa-miR-3120-5p | | 2.13465309 | | 7.89E-10 | | UP |
| hsa-miR-532-3p | | -1.081785158 | | 0.00700589 | | DOWN |
| hsa-miR-146a-5p | | 1.420776953 | | 1.67E-10 | | UP |
| hsa-miR-4738-3p | | 4.077698177 | | 0.001198069 | | UP |
| hsa-miR-342-5p | | -1.086411172 | | 1.13E-06 | | DOWN |
| hsa-miR-134-5p | | 1.09270934 | | 3.50E-06 | | UP |
| hsa-miR-206 | | -2.082773177 | | 1.47E-17 | | DOWN |
| hsa-miR-1248 | | -1.883989878 | | 0.006038783 | | DOWN |
| hsa-miR-200b-5p | | -1.365113125 | | 0.00025716 | | DOWN |
| hsa-miR-548j-5p | | 1.985971846 | | 3.52E-17 | | UP |
| hsa-miR-141-3p | | -3.112341153 | | 3.00E-16 | | DOWN |
| hsa-miR-4665-5p | | 1.036639182 | | 0.001401291 | | UP |
| hsa-miR-483-5p | | -1.301339822 | | 1.66E-07 | | DOWN |
| hsa-miR-615-3p | | -2.89036097 | | 1.27E-29 | | DOWN |
| hsa-miR-378c | | -1.078738629 | | 1.77E-05 | | DOWN |
| hsa-miR-151a-3p | | 1.108603732 | | 5.69E-07 | | UP |
| hsa-miR-548o-3p | | 1.709798418 | | 3.35E-10 | | UP |
| hsa-miR-6741-5p | | -1.100893649 | | 0.004210745 | | DOWN |
| hsa-miR-338-3p | | -2.495981482 | | 1.31E-07 | | DOWN |
| hsa-miR-1277-5p | | 5.991903252 | | 2.18E-11 | | UP |
| hsa-miR-215-5p | | -3.157663893 | | 4.47E-40 | | DOWN |
| hsa-miR-200c-3p | | -1.068861683 | | 5.07E-06 | | DOWN |
| hsa-miR-130b-3p | | -1.401859882 | | 6.78E-05 | | DOWN |
| hsa-miR-326 | | 1.342279163 | | 0.000364148 | | UP |
| hsa-miR-6721-5p | | 1.293657753 | | 7.83E-05 | | UP |
| hsa-miR-125b-1-3p | | -2.98676406 | | 4.00E-12 | | DOWN |
| hsa-miR-323b-3p | | 2.548184969 | | 2.27E-15 | | UP |
| hsa-miR-30c-2-3p | | -1.462728619 | | 5.70E-06 | | DOWN |
| hsa-miR-514a-3p | | -4.61393309 | | 1.96E-33 | | DOWN |
| hsa-miR-584-5p | | 1.01412038 | | 6.01E-06 | | UP |
| hsa-miR-1260b | | 5.425647106 | | 1.57E-07 | | UP |
| hsa-miR-605-3p | | 1.596526684 | | 0.037217673 | | UP |
| hsa-miR-340-3p | | 1.120191745 | | 1.13E-06 | | UP |
| hsa-miR-132-5p | | -1.313386542 | | 2.37E-05 | | DOWN |
| hsa-miR-1271-5p | | -1.055950221 | | 0.009662663 | | DOWN |
| hsa-miR-494-3p | | 2.623260445 | | 2.20E-16 | | UP |
| hsa-miR-516b-5p | | -1.051983573 | | 0.004900348 | | DOWN |
| hsa-miR-96-5p | | -1.359174829 | | 5.88E-06 | | DOWN |
| hsa-miR-3157-3p | | -2.117701179 | | 1.87E-06 | | DOWN |
| hsa-miR-24-1-5p | | -1.28241055 | | 7.67E-05 | | DOWN |
| hsa-miR-3120-3p | | 1.928610266 | | 2.63E-06 | | UP |
| hsa-miR-429 | | -3.434111257 | | 2.46E-24 | | DOWN |
| hsa-miR-455-5p | | -2.436557842 | | 1.81E-17 | | DOWN |
| hsa-miR-4742-5p | | 1.013350176 | | 0.005024414 | | UP |
| hsa-miR-365a-3p | | -1.871115839 | | 1.75E-08 | | DOWN |
| hsa-miR-668-3p | | 1.655626919 | | 0.012489285 | | UP |
| hsa-miR-5010-3p | | -1.527954973 | | 6.43E-07 | | DOWN |
| hsa-miR-92b-3p | | -1.293021231 | | 5.65E-08 | | DOWN |
| hsa-miR-4659b-3p | | 2.082455187 | | 1.94E-06 | | UP |
| hsa-miR-6861-5p | | -2.374508254 | | 0.01052506 | | DOWN |
| hsa-miR-200b-3p | | -2.128460531 | | 1.32E-19 | | DOWN |
| hsa-miR-1228-5p | | -1.805100186 | | 1.48E-10 | | DOWN |
| hsa-miR-98-5p | | 1.404719944 | | 3.47E-10 | | UP |
| hsa-miR-218-5p | | -2.599002549 | | 5.86E-21 | | DOWN |
| hsa-miR-660-5p | | -1.056801189 | | 0.000173342 | | DOWN |
| hsa-miR-146a-3p | | 2.067991001 | | 6.41E-10 | | UP |
| hsa-miR-301a-5p | | 1.103910785 | | 0.000730665 | | UP |
| hsa-miR-375 | | -1.86253363 | | 4.00E-17 | | DOWN |
| hsa-miR-9-3p | | -2.823422036 | | 1.98E-17 | | DOWN |
| hsa-miR-1255a | | 2.249694619 | | 1.39E-08 | | UP |
| hsa-miR-184 | | -1.320014529 | | 2.71E-06 | | DOWN |
| hsa-miR-28-5p | | 1.211459867 | | 1.55E-07 | | UP |
| hsa-miR-3187-3p | | 1.950513514 | | 0.000379083 | | UP |
| hsa-miR-196a-5p | | -3.786760882 | | 6.26E-34 | | DOWN |
| hsa-miR-224-5p | | 1.07814716 | | 1.65E-06 | | UP |
| hsa-miR-125b-5p | | -1.147100803 | | 3.09E-07 | | DOWN |
| hsa-miR-3138 | | 1.899325595 | | 0.000132948 | | UP |
| hsa-miR-124-3p | | -5.692067808 | | 2.51E-19 | | DOWN |
| hsa-let-7c-5p | | -1.298212495 | | 5.04E-09 | | DOWN |
| hsa-miR-100-5p | | -1.415115793 | | 3.15E-10 | | DOWN |
| hsa-miR-6862-5p | | 1.543879274 | | 1.99E-05 | | UP |
| hsa-miR-708-3p | | -2.787542608 | | 7.94E-17 | | DOWN |
| hsa-miR-150-3p | | -1.366310148 | | 3.93E-09 | | DOWN |
| hsa-miR-517b-3p | | -2.045136273 | | 0.006618568 | | DOWN |
| hsa-miR-550a-3-5p | | -1.097457089 | | 0.000287425 | | DOWN |
| hsa-miR-651-5p | | -1.001508011 | | 0.012590893 | | DOWN |
| hsa-miR-641 | | 1.864711678 | | 2.05E-09 | | UP |
| hsa-miR-204-5p | | -1.349209707 | | 1.42E-08 | | DOWN |
| hsa-miR-451a | | -1.167701893 | | 1.36E-07 | | DOWN |
| hsa-miR-493-5p | | 2.271817274 | | 2.26E-17 | | UP |
| hsa-miR-1323 | | 1.859819434 | | 6.98E-07 | | UP |
| hsa-miR-7848-3p | | 1.247366931 | | 0.001046139 | | UP |
| hsa-miR-421 | | 1.144402444 | | 1.69E-06 | | UP |
| hsa-miR-628-3p | | 1.384709486 | | 1.23E-07 | | UP |
| hsa-miR-486-5p | | -1.533122376 | | 4.08E-12 | | DOWN |
| hsa-miR-369-5p | | 1.674996834 | | 7.61E-10 | | UP |
| hsa-miR-889-3p | | 2.280587964 | | 2.11E-09 | | UP |
| hsa-miR-103a-3p | | 1.004297691 | | 6.29E-06 | | UP |
| hsa-miR-335-3p | | 1.635163462 | | 4.00E-12 | | UP |
| hsa-miR-431-5p | | 1.42831668 | | 0.000143387 | | UP |
| hsa-miR-506-3p | | -1.66718607 | | 0.007844767 | | DOWN |
| hsa-miR-379-3p | | 2.073839314 | | 0.000104989 | | UP |
| hsa-miR-4467 | | -1.636640584 | | 2.64E-06 | | DOWN |
| hsa-miR-509-3-5p | | -3.092340157 | | 2.71E-19 | | DOWN |
| hsa-miR-212-5p | | -2.37624052 | | 4.18E-10 | | DOWN |
| hsa-miR-487a-5p | | 1.278974566 | | 0.028970257 | | UP |
| hsa-miR-3613-5p | | 1.018809259 | | 0.000115088 | | UP |
| hsa-miR-629-3p | | -1.725524456 | | 9.02E-06 | | DOWN |
| hsa-miR-214-3p | | -1.880713918 | | 9.24E-10 | | DOWN |
| hsa-miR-129-5p | | -2.021863568 | | 1.68E-15 | | DOWN |
| hsa-miR-192-5p | | -1.372637774 | | 6.87E-10 | | DOWN |
| hsa-miR-92b-5p | | -1.313368842 | | 3.55E-07 | | DOWN |
| hsa-miR-145-5p | | -1.818758747 | | 6.50E-09 | | DOWN |
| hsa-miR-193b-5p | | -1.534675107 | | 1.69E-09 | | DOWN |
| hsa-miR-1306-5p | | -2.679568089 | | 5.02E-13 | | DOWN |
| hsa-miR-654-3p | | 1.183310949 | | 1.14E-06 | | UP |
| hsa-miR-487b-3p | | 2.017102174 | | 6.02E-10 | | UP |
| hsa-miR-202-3p | | -1.149037583 | | 0.010674186 | | DOWN |
| hsa-miR-329-3p | | 2.14087688 | | 1.13E-14 | | UP |
| hsa-miR-508-3p | | -2.533618579 | | 0.000220841 | | DOWN |
| hsa-miR-1-3p | | -3.750310404 | | 1.11E-57 | | DOWN |
| hsa-miR-4732-3p | | -1.980159315 | | 2.20E-16 | | DOWN |
| hsa-miR-3158-3p | | -1.242118558 | | 1.59E-07 | | DOWN |
| hsa-miR-1303 | | -1.817894468 | | 1.90E-06 | | DOWN |
| hsa-miR-122-5p | | -1.203468319 | | 5.65E-08 | | DOWN |
| hsa-miR-486-3p | | -1.139961774 | | 3.14E-07 | | DOWN |
| hsa-miR-93-3p | | 1.020905841 | | 2.62E-05 | | UP |
| hsa-miR-493-3p | | 1.590214681 | | 1.39E-09 | | UP |
| hsa-miR-185-5p | | 1.062558184 | | 1.65E-06 | | UP |
| hsa-miR-200a-5p | | -2.880586999 | | 6.12E-18 | | DOWN |
| hsa-miR-3934-5p | | -3.376881669 | | 6.76E-08 | | DOWN |
| hsa-miR-204-3p | | -1.01459354 | | 0.003693229 | | DOWN |
| hsa-miR-26a-5p | | 1.139900985 | | 2.71E-07 | | UP |
| hsa-miR-199a-5p | | 1.195224475 | | 1.05E-07 | | UP |
| hsa-miR-576-3p | | 1.401418323 | | 3.84E-08 | | UP |
| hsa-miR-150-5p | | -1.377061055 | | 6.41E-10 | | DOWN |
| hsa-miR-642a-3p | | -1.396687714 | | 3.41E-07 | | DOWN |
| hsa-miR-365b-5p | | -2.157410133 | | 2.54E-06 | | DOWN |
| hsa-miR-221-3p | | 1.007433412 | | 5.88E-06 | | UP |
| hsa-miR-378a-5p | | -1.030745682 | | 0.000307929 | | DOWN |
| hsa-miR-6847-5p | | 3.500404915 | | 2.72E-08 | | UP |
| hsa-miR-4685-3p | | -1.964511817 | | 3.28E-06 | | DOWN |
| hsa-miR-194-5p | | -1.442325423 | | 1.25E-10 | | DOWN |
| hsa-miR-4654 | | 2.123270084 | | 0.000242361 | | UP |
| hsa-miR-335-5p | | 1.643497445 | | 8.98E-10 | | UP |
| hsa-let-7b-3p | | -1.016726201 | | 4.55E-05 | | DOWN |
| hsa-miR-9-5p | | -3.844061644 | | 1.76E-54 | | DOWN |
| **DEmiRs in trAMWoA before and after TA treatment** | | | | | | |
| hsa-miR-625-5p | -1.22807287 | | 0.002593839 | | DOWN | |
| hsa-miR-4732-5p | 1.134468225 | | 0.002602065 | | UP | |
| hsa-miR-1285-3p | 1.536823857 | | 0.002137269 | | UP | |
| hsa-miR-1185-1-3p | 1.724673511 | | 0.003554682 | | UP | |
| hsa-miR-548k | 1.868799919 | | 0.008364887 | | UP | |
| hsa-miR-1292-5p | 1.097089132 | | 0.027271365 | | UP | |
| hsa-miR-5010-5p | 1.30546236 | | 0.005738252 | | UP | |
| hsa-miR-378c | 1.001024298 | | 0.032508135 | | UP | |
| hsa-miR-6516-5p | 1.511863055 | | 0.001326274 | | UP | |
| hsa-miR-605-3p | -1.178244445 | | 0.033331637 | | DOWN | |
| hsa-miR-5010-3p | 1.365933042 | | 0.022722975 | | UP | |
| hsa-miR-660-5p | 1.089073664 | | 0.019279979 | | UP | |
| hsa-miR-375 | 1.054776396 | | 0.000536334 | | UP | |
| hsa-miR-6862-5p | -2.802895586 | | 1.72E-05 | | DOWN | |
| hsa-miR-550a-3-5p | 1.609698484 | | 0.000230076 | | UP | |
| hsa-miR-6515-5p | 1.067545307 | | 0.020853197 | | UP | |
| hsa-miR-6837-3p | 1.802805181 | | 0.011265874 | | UP | |
| hsa-miR-451a | 1.298799574 | | 4.48E-06 | | UP | |
| hsa-miR-369-5p | -1.530297359 | | 5.55E-05 | | DOWN | |
| hsa-miR-29b-3p | 1.219068109 | | 0.003286832 | | UP | |
| hsa-miR-143-5p | -3.915801509 | | 6.57E-07 | | DOWN | |
| hsa-let-7f-1-3p | 1.374494358 | | 0.00892526 | | UP | |
| hsa-miR-151b | -2.366345668 | | 0.000228348 | | DOWN | |
| hsa-miR-3173-5p | -1.115790947 | | 0.032351918 | | DOWN | |
| hsa-miR-145-5p | 2.42012439 | | 1.57E-11 | | UP | |
| hsa-miR-501-3p | 1.041953316 | | 0.002052775 | | UP | |
| hsa-miR-20b-5p | 1.590137037 | | 5.55E-05 | | UP | |
| hsa-miR-10b-3p | -1.231923847 | | 0.003316704 | | DOWN | |
| hsa-miR-378a-5p | 1.647392696 | | 0.001682791 | | UP | |
| hsa-miR-4654 | -1.163072043 | | 0.01899495 | | DOWN | |
| **DEmiRs in shAMWoA before and after SA treatment** | | | | | | |
| hsa-miR-518f-5p | 1.62143301 | | 0.01850591 | | UP | |
| hsa-miR-410-3p | 6.465319014 | | 6.05E-11 | | UP | |
| hsa-miR-449a | -6.761268223 | | 5.73E-14 | | DOWN | |
| hsa-miR-3064-5p | -2.460071618 | | 1.86E-06 | | DOWN | |
| hsa-miR-147b | -1.329333095 | | 0.000426833 | | DOWN | |
| hsa-miR-500a-3p | -1.71925404 | | 0.001028045 | | DOWN | |
| hsa-miR-4732-5p | -1.45106356 | | 0.009978487 | | DOWN | |
| hsa-miR-4659a-3p | 5.774417836 | | 3.31E-07 | | UP | |
| hsa-miR-542-3p | -1.036810693 | | 0.000127804 | | DOWN | |
| hsa-miR-2355-3p | 6.24246641 | | 4.91E-09 | | UP | |
| hsa-miR-450a-5p | 1.125651262 | | 0.000107124 | | UP | |
| hsa-miR-365b-3p | 1.755696827 | | 0.000457696 | | UP | |
| hsa-miR-541-3p | -1.635943069 | | 0.003583297 | | DOWN | |
| hsa-miR-4485-3p | 4.810420617 | | 4.86E-05 | | UP | |
| hsa-miR-409-5p | 1.200402231 | | 0.001658655 | | UP | |
| hsa-miR-25-5p | -2.439085667 | | 0.000612655 | | DOWN | |
| hsa-miR-149-5p | -1.089518731 | | 0.00156411 | | DOWN | |
| hsa-miR-1278 | 5.589421112 | | 9.25E-06 | | UP | |
| hsa-miR-760 | -3.500349624 | | 0.007047182 | | DOWN | |
| hsa-miR-526a | 1.62143301 | | 0.01850591 | | UP | |
| hsa-miR-592 | -2.344297164 | | 7.67E-10 | | DOWN | |
| hsa-miR-363-5p | 6.29434643 | | 4.79E-08 | | UP | |
| hsa-miR-548av-3p | 7.288706861 | | 6.38E-13 | | UP | |
| hsa-miR-1292-5p | -1.248227604 | | 0.007061622 | | DOWN | |
| hsa-miR-26a-2-3p | 2.903525626 | | 0.002563642 | | UP | |
| hsa-miR-4662a-5p | 3.046102258 | | 0.000133338 | | UP | |
| hsa-miR-1273h-5p | -6.197297487 | | 1.83E-09 | | DOWN | |
| hsa-miR-4664-3p | -5.537096706 | | 4.05E-06 | | DOWN | |
| hsa-miR-141-3p | 2.535175844 | | 6.86E-15 | | UP | |
| hsa-miR-30d-3p | 2.631344193 | | 4.42E-13 | | UP | |
| hsa-miR-5683 | 2.779317214 | | 0.020480631 | | UP | |
| hsa-miR-483-5p | 1.258639828 | | 0.004956113 | | UP | |
| hsa-miR-125b-2-3p | -1.409039376 | | 0.000223968 | | DOWN | |
| hsa-miR-338-3p | 1.653788327 | | 4.86E-05 | | UP | |
| hsa-miR-130b-3p | 1.749525648 | | 0.006081384 | | UP | |
| hsa-miR-454-5p | -1.1471204 | | 0.032938173 | | DOWN | |
| hsa-miR-182-3p | -1.957757302 | | 2.99E-08 | | DOWN | |
| hsa-miR-518d-5p | 1.62143301 | | 0.01850591 | | UP | |
| hsa-miR-30c-2-3p | -1.780811339 | | 6.59E-05 | | DOWN | |
| hsa-miR-505-5p | 1.303641538 | | 0.004462135 | | UP | |
| hsa-miR-411-3p | 1.635278911 | | 0.001397016 | | UP | |
| hsa-miR-153-3p | -1.708886186 | | 3.25E-05 | | DOWN | |
| hsa-miR-1288-3p | 3.856617401 | | 2.45E-07 | | UP | |
| hsa-miR-494-3p | 1.10071655 | | 0.001075783 | | UP | |
| hsa-miR-92a-1-5p | -1.769433356 | | 7.83E-05 | | DOWN | |
| hsa-miR-4521 | 4.113524135 | | 2.37E-10 | | UP | |
| hsa-miR-3120-3p | 1.128656731 | | 0.017129832 | | UP | |
| hsa-miR-191-3p | 2.507825412 | | 0.003985302 | | UP | |
| hsa-miR-148b-5p | -1.511286953 | | 0.023601312 | | DOWN | |
| hsa-miR-365a-3p | 1.755696827 | | 0.000457696 | | UP | |
| hsa-miR-590-3p | 5.857122515 | | 3.31E-07 | | UP | |
| hsa-miR-511-5p | -1.213831626 | | 0.002878316 | | DOWN | |
| hsa-miR-660-5p | 2.413040188 | | 2.15E-08 | | UP | |
| hsa-miR-146a-3p | 3.321748596 | | 5.08E-11 | | UP | |
| hsa-miR-4661-5p | 2.273871771 | | 0.00010772 | | UP | |
| hsa-miR-766-5p | 2.985320762 | | 1.21E-05 | | UP | |
| hsa-miR-3138 | -1.968699444 | | 0.000243285 | | DOWN | |
| hsa-miR-504-5p | -1.017339255 | | 0.006310579 | | DOWN | |
| hsa-miR-3190-3p | 5.888602363 | | 7.72E-07 | | UP | |
| hsa-miR-520c-5p | 1.62143301 | | 0.01850591 | | UP | |
| hsa-miR-15b-3p | 1.044059647 | | 0.000526958 | | UP | |
| hsa-miR-6515-5p | 1.839312952 | | 0.000223968 | | UP | |
| hsa-let-7e-3p | -3.512663418 | | 0.002878316 | | DOWN | |
| hsa-miR-135a-5p | -1.244430364 | | 8.16E-06 | | DOWN | |
| hsa-miR-30c-1-3p | 1.302505319 | | 0.006081384 | | UP | |
| hsa-miR-628-3p | 2.421735075 | | 4.25E-08 | | UP | |
| hsa-miR-30b-3p | 6.002912678 | | 6.98E-08 | | UP | |
| hsa-miR-7976 | -2.936863507 | | 2.77E-05 | | DOWN | |
| hsa-miR-137 | -3.946770943 | | 1.83E-09 | | DOWN | |
| hsa-miR-27b-5p | 3.111752489 | | 1.54E-09 | | UP | |
| hsa-miR-193b-5p | 6.647015626 | | 1.90E-09 | | UP | |
| hsa-miR-548e-3p | 1.244378269 | | 0.002747509 | | UP | |
| hsa-miR-124-5p | -1.25329039 | | 2.63E-06 | | DOWN | |
| hsa-miR-378d | 1.160824031 | | 0.006081384 | | UP | |
| hsa-miR-101-5p | -6.997075309 | | 6.38E-13 | | DOWN | |
| hsa-miR-6855-5p | 6.453207369 | | 1.83E-09 | | UP | |
| hsa-miR-1304-5p | 2.950411386 | | 0.001279918 | | UP | |
| hsa-miR-1908-5p | 1.517393847 | | 2.39E-05 | | UP | |
| hsa-miR-502-3p | -1.079331185 | | 0.038113002 | | DOWN | |
| hsa-miR-4765 | 5.772496519 | | 1.02E-07 | | UP | |
| hsa-miR-20b-5p | 1.273970545 | | 0.043616665 | | UP | |
| hsa-miR-10b-3p | -2.683034056 | | 2.97E-05 | | DOWN | |
| hsa-miR-15a-5p | -1.275051445 | | 0.007047182 | | DOWN | |
| hsa-miR-7151-5p | -2.232601827 | | 3.81E-05 | | DOWN | |
| hsa-miR-369-3p | -1.007742167 | | 0.00724742 | | DOWN | |
| **DEmiRs in HC before and after TA treatment** | | | | | | |
| hsa-miR-503-5p | 1.798852528 | | 0.000150072 | | UP | |
| hsa-miR-1283 | -4.732563986 | | 0.001189278 | | DOWN | |
| hsa-miR-4647 | 4.971990025 | | 1.44E-05 | | UP | |
| hsa-miR-939-5p | 2.834158967 | | 3.93E-05 | | UP | |
| hsa-miR-147b | -1.239067997 | | 0.015878501 | | DOWN | |
| hsa-miR-3074-5p | -5.097953216 | | 0.000151457 | | DOWN | |
| hsa-miR-18a-3p | 2.712600515 | | 2.15E-05 | | UP | |
| hsa-miR-4732-5p | -1.149650948 | | 0.001925814 | | DOWN | |
| hsa-miR-34c-5p | -5.902922251 | | 1.98E-07 | | DOWN | |
| hsa-miR-454-3p | -2.423877172 | | 0.00057974 | | DOWN | |
| hsa-miR-542-3p | 1.702802949 | | 0.000875308 | | UP | |
| hsa-miR-381-3p | 1.397369069 | | 5.86E-06 | | UP | |
| hsa-miR-1226-5p | -4.762890184 | | 9.64E-05 | | DOWN | |
| hsa-miR-6767-5p | -4.544343128 | | 0.00180335 | | DOWN | |
| hsa-miR-365b-3p | -1.349283974 | | 0.003226973 | | DOWN | |
| hsa-miR-136-5p | -1.116310957 | | 0.000536271 | | DOWN | |
| hsa-miR-382-3p | 5.593603614 | | 5.02E-07 | | UP | |
| hsa-miR-25-5p | -1.075716864 | | 0.003270544 | | DOWN | |
| hsa-miR-149-5p | -1.804496429 | | 0.018765526 | | DOWN | |
| hsa-miR-363-5p | -2.612831027 | | 0.001861217 | | DOWN | |
| hsa-miR-6503-3p | 4.853966947 | | 3.93E-05 | | UP | |
| hsa-miR-548k | -5.203702325 | | 1.27E-05 | | DOWN | |
| hsa-miR-4662a-5p | 5.321436329 | | 2.90E-07 | | UP | |
| hsa-miR-365a-5p | -1.666327637 | | 0.00189034 | | DOWN | |
| hsa-miR-23b-5p | -2.546280062 | | 1.85E-08 | | DOWN | |
| hsa-miR-17-3p | -3.398718193 | | 0.00091098 | | DOWN | |
| hsa-miR-148b-3p | 1.06498095 | | 0.00051933 | | UP | |
| hsa-miR-199b-5p | 2.809719788 | | 9.25E-08 | | UP | |
| hsa-miR-1255b-5p | 3.534162439 | | 2.45E-08 | | UP | |
| hsa-miR-6511a-3p | -1.27236307 | | 0.033629995 | | DOWN | |
| hsa-miR-532-3p | -2.216677043 | | 0.002342731 | | DOWN | |
| hsa-miR-206 | -1.443428307 | | 1.65E-05 | | DOWN | |
| hsa-miR-31-5p | 1.948718217 | | 0.000536271 | | UP | |
| hsa-miR-1248 | 1.297179874 | | 0.001202794 | | UP | |
| hsa-miR-200b-5p | -3.637309782 | | 2.90E-07 | | DOWN | |
| hsa-miR-5010-5p | 1.81987912 | | 0.010005647 | | UP | |
| hsa-miR-141-3p | -1.041842088 | | 0.02125099 | | DOWN | |
| hsa-miR-219a-2-3p | -7.899442296 | | 4.22E-23 | | DOWN | |
| hsa-miR-330-5p | -2.682593742 | | 1.43E-07 | | DOWN | |
| hsa-miR-615-3p | -3.702712641 | | 1.34E-20 | | DOWN | |
| hsa-miR-424-3p | -1.170888769 | | 0.000568269 | | DOWN | |
| hsa-miR-23a-5p | -1.623553468 | | 4.71E-05 | | DOWN | |
| hsa-miR-548o-3p | 2.245635951 | | 3.75E-05 | | UP | |
| hsa-miR-32-5p | 1.148036099 | | 0.001196605 | | UP | |
| hsa-miR-338-3p | -3.13735952 | | 7.00E-08 | | DOWN | |
| hsa-miR-193a-5p | -1.01538935 | | 0.001475343 | | DOWN | |
| hsa-miR-136-3p | -4.579655217 | | 0.002692559 | | DOWN | |
| hsa-miR-200c-3p | -1.490111236 | | 2.65E-05 | | DOWN | |
| hsa-miR-144-3p | 2.06076837 | | 3.28E-10 | | UP | |
| hsa-miR-485-3p | 1.245936241 | | 0.000954652 | | UP | |
| hsa-miR-130b-3p | 2.226168002 | | 0.023618671 | | UP | |
| hsa-miR-326 | 1.663982266 | | 0.001134087 | | UP | |
| hsa-miR-4781-3p | -4.832526724 | | 0.000537861 | | DOWN | |
| hsa-miR-454-5p | 1.44235523 | | 0.002466744 | | UP | |
| hsa-miR-125b-1-3p | -2.37654987 | | 6.02E-05 | | DOWN | |
| hsa-miR-323b-3p | 1.979656259 | | 0.000197558 | | UP | |
| hsa-miR-30c-2-3p | -1.451394457 | | 0.000470014 | | DOWN | |
| hsa-miR-514a-3p | -2.797296918 | | 5.77E-07 | | DOWN | |
| hsa-miR-505-5p | -1.045375868 | | 0.000443656 | | DOWN | |
| hsa-miR-21-3p | 1.94815826 | | 0.025202615 | | UP | |
| hsa-miR-494-3p | 2.172217671 | | 1.65E-05 | | UP | |
| hsa-miR-92a-1-5p | 1.122766363 | | 0.001734603 | | UP | |
| hsa-miR-516b-5p | -1.735012557 | | 5.58E-06 | | DOWN | |
| hsa-miR-96-5p | -1.125391474 | | 0.000559553 | | DOWN | |
| hsa-miR-3157-3p | -2.551392591 | | 0.004397914 | | DOWN | |
| hsa-miR-5193 | -1.139500587 | | 0.018765526 | | DOWN | |
| hsa-miR-24-1-5p | -1.810158696 | | 1.51E-05 | | DOWN | |
| hsa-miR-3120-3p | 1.841124281 | | 0.018390578 | | UP | |
| hsa-miR-455-5p | -1.713421069 | | 0.000144653 | | DOWN | |
| hsa-miR-145-3p | -1.486133625 | | 5.58E-06 | | DOWN | |
| hsa-miR-365a-3p | -1.349283974 | | 0.003226973 | | DOWN | |
| hsa-miR-210-3p | 1.702068487 | | 0.004384236 | | UP | |
| hsa-miR-1228-5p | -2.87059388 | | 6.17E-08 | | DOWN | |
| hsa-miR-218-5p | -2.55111447 | | 4.22E-11 | | DOWN | |
| hsa-miR-197-5p | -4.543811249 | | 0.00180335 | | DOWN | |
| hsa-miR-324-5p | -1.956843662 | | 0.000954652 | | DOWN | |
| hsa-miR-574-3p | -1.629654453 | | 1.02E-06 | | DOWN | |
| hsa-miR-301a-5p | 2.417527879 | | 7.00E-08 | | UP | |
| hsa-miR-9-3p | -2.494430884 | | 1.74E-05 | | DOWN | |
| hsa-miR-184 | 1.052396487 | | 0.001794875 | | UP | |
| hsa-miR-29c-3p | -1.21669213 | | 0.038245379 | | DOWN | |
| hsa-miR-101-3p | 1.100676604 | | 0.000197558 | | UP | |
| hsa-miR-345-5p | -1.430091321 | | 0.000156942 | | DOWN | |
| hsa-miR-196a-5p | -3.023204448 | | 1.50E-13 | | DOWN | |
| hsa-miR-372-3p | 2.557811669 | | 0.002431296 | | UP | |
| hsa-miR-3138 | 5.068663722 | | 6.63E-05 | | UP | |
| hsa-miR-124-3p | -6.597013239 | | 2.13E-11 | | DOWN | |
| hsa-let-7c-5p | -1.639571147 | | 2.60E-08 | | DOWN | |
| hsa-miR-576-5p | 1.671033275 | | 0.001202794 | | UP | |
| hsa-miR-127-5p | -5.30483607 | | 4.00E-05 | | DOWN | |
| hsa-miR-641 | 1.217856301 | | 0.020523298 | | UP | |
| hsa-miR-451a | 1.136864531 | | 8.80E-05 | | UP | |
| hsa-miR-493-5p | 1.778370158 | | 2.86E-06 | | UP | |
| hsa-miR-1298-5p | -2.429369937 | | 0.014155492 | | DOWN | |
| hsa-miR-421 | 2.183895983 | | 4.40E-05 | | UP | |
| hsa-miR-628-3p | 1.145630264 | | 0.002400662 | | UP | |
| hsa-miR-7976 | -1.214810997 | | 0.020148952 | | DOWN | |
| hsa-miR-143-5p | -1.574184845 | | 0.004079581 | | DOWN | |
| hsa-miR-509-3-5p | -6.830879865 | | 2.73E-13 | | DOWN | |
| hsa-miR-212-5p | -2.358444924 | | 0.0003276 | | DOWN | |
| hsa-miR-6858-5p | 5.917395332 | | 3.28E-10 | | UP | |
| hsa-miR-6842-5p | -1.706614925 | | 0.000947042 | | DOWN | |
| hsa-miR-214-3p | -1.373866463 | | 0.001655526 | | DOWN | |
| hsa-miR-129-5p | -1.227000261 | | 0.000391407 | | DOWN | |
| hsa-miR-130a-3p | -2.777720126 | | 2.34E-05 | | DOWN | |
| hsa-miR-664a-5p | -1.711937866 | | 2.60E-08 | | DOWN | |
| hsa-miR-1299 | -2.494562071 | | 0.046546973 | | DOWN | |
| hsa-miR-145-5p | -1.409990153 | | 0.000160957 | | DOWN | |
| hsa-miR-193b-5p | -2.292154919 | | 2.34E-07 | | DOWN | |
| hsa-miR-1306-5p | -5.997102754 | | 1.79E-08 | | DOWN | |
| hsa-miR-487b-3p | 1.554945019 | | 0.002581062 | | UP | |
| hsa-miR-329-3p | 6.636625473 | | 2.97E-13 | | UP | |
| hsa-miR-1-3p | -1.080464099 | | 0.000315054 | | DOWN | |
| hsa-miR-378d | 1.10092723 | | 0.016645646 | | UP | |
| hsa-miR-29c-5p | -3.890270828 | | 2.90E-07 | | DOWN | |
| hsa-miR-6770-3p | 5.18424857 | | 2.31E-05 | | UP | |
| hsa-miR-122-5p | -1.102839464 | | 0.000197558 | | DOWN | |
| hsa-miR-502-3p | -1.290179014 | | 0.016578712 | | DOWN | |
| hsa-miR-1291 | 4.825795297 | | 9.67E-42 | | UP | |
| hsa-miR-3605-5p | -1.972448304 | | 0.000914982 | | DOWN | |
| hsa-miR-374a-5p | 1.762144364 | | 1.96E-06 | | UP | |
| hsa-miR-642a-3p | -1.258417125 | | 0.00189034 | | DOWN | |
| hsa-miR-1249-3p | 1.732308703 | | 0.000905872 | | UP | |
| hsa-miR-758-3p | -1.152302696 | | 0.000875308 | | DOWN | |
| hsa-miR-4683 | -1.758870391 | | 0.001098912 | | DOWN | |
| hsa-miR-125a-3p | -1.530303268 | | 0.000580231 | | DOWN | |
| hsa-miR-7854-3p | 2.331255572 | | 0.000166817 | | UP | |
| hsa-miR-19b-3p | 1.902667523 | | 0.001652959 | | UP | |
| hsa-miR-7-1-3p | -4.597746364 | | 0.002692559 | | DOWN | |

Notes: DEmiRs, differentially expressed miRNAs; MWoA, migraine without aura; TA, true acupuncture; SA, sham acupuncture; HC, healthy control; trAMWoA, true acupuncture group; shAMWoA, sham acupuncture group
